# Determination of Quantitative Trait Loci (QTL) for Early Maturation in Rainbow Trout (*Oncorhynchus mykiss*)

**DOI:** 10.1007/s10126-008-9098-5

**Published:** 2008-05-20

**Authors:** Lisa Haidle, Jennifer E. Janssen, Karim Gharbi, Hooman K. Moghadam, Moira M. Ferguson, Roy G. Danzmann

**Affiliations:** 1 Department of Integrative Biology, University of Guelph, Guelph, ON Canada N1G 2W1; 2 Present Address: Institute of Comparative Medicine, University of Glasgow, Glasgow, UK G61 1QH

**Keywords:** QTL, Early maturation, Salmonids, Life history

## Abstract

To identify quantitative trait loci (QTL) influencing early maturation (EM) in rainbow trout (*Oncorhynchus mykiss*), a genome scan was performed using 100 microsatellite loci across 29 linkage groups. Six inter-strain paternal half-sib families using three inter-strain F_1_ brothers (approximately 50 progeny in each family) derived from two strains that differ in the propensity for EM were used in the study. Alleles derived from both parental sources were observed to contribute to the expression of EM in the progeny of the brothers. Four genome-wide significant QTL regions (i.e., RT-8, -17, -24, and -30) were observed. EM QTL detected on RT-8 and -24 demonstrated significant and suggestive QTL effects in both male and female progeny. Furthermore, within both male and female full-sib groupings, QTL on RT-8 and -24 were detected in two or more of the five parents used. Significant genome-wide and several strong chromosome-wide QTL for EM localized to different regions in males and females, suggesting some sex-specific control. Namely, QTL detected on RT-13, -15, -21, and -30 were associated with EM only in females, and those on RT-3, -17, and -19 were associated with EM only in males. Within the QTL regions identified, a comparison of syntenic EST markers from the rainbow trout linkage map with the zebrafish (*Danio rerio*) genome identified several putative candidate genes that may influence EM.

**Electronic supplementary material:**

The online version of this article (doi:10.1007/s10126-008-9098-5) contains supplementary material, which is available to authorized users.

## Introduction

The regulation of sexual maturation in vertebrates is a complex process that is determined by both genetic and epigenetic factors conditioned by sex-specific intrinsic cellular conditions as well as extrinsic environmental factors (e.g., food availability). For example, chromatin modifications such as differential gonadal chromatin methylation occur at early stages in human development in a sex-specific manner, and such modifications persist as stable heritable characteristics across generations (Galetzka et al. 2007; Adcock and Lee 2006). Physiologically, sex-specific background also mediates the expression of other performance and life-history traits. In model organisms such as the nematode *Caenorhabditis elegans*, *Drosophila*, humans, and mice, it has been shown that it is not unusual for some genes and somatic tissues to display sexually dimorphic expression and reactions to hormones (reviewed by Rinn and Snyder 2005). Developmental gene expression is differentially regulated between the sexes in *C. elegans* (Jiang et al. 2001) and molecular polymorphisms in candidate genes affecting number of sensory bristles are highly sex-specific in *Drosophila* (Lyman et al. 1999). Quantitative trait loci (QTL) for longevity also have been seen to be highly sex-specific in both *Drosophila* (Nuzhdin et al. 1997) and humans (De Benedictis et al. 1998; Varcasia et al. 2001). Moreover, in mice, two sets of body weight QTL have been located that are either sex-specific or generic (Vaughn et al. 1999).

Maturation timing is part of the complex life history of salmonids and has environmental and genetic components (Marschall et al. 1998; Thorpe and Metcalfe 1998). Maturation can occur prematurely if fish weights and lipid reserves exceed certain ‘physiological thresholds for maturation’ (Thorpe and Metcalfe 1998). It is also well known that female and male salmonids display differential rates of early maturation (EM) (Naevdal 1983; Johnstone 1993; Devlin and Nagahama 2002). Although the underlying genetic causes have not been well studied, a strong genetic correlation has been detected in age of maturation between the sexes (Kause et al. 2003). These researchers concluded, however, that the evidence was minimal for the differential influence of genes affecting maturation in either sex. However, while a strong genetic correlation exists in age of maturation between the sexes, without further research into the molecular mechanisms underlying this complex trait it cannot be concluded unequivocally that generic EM QTL will regulate sexual maturation uniformly in both sexes.

In salmonids, life-history traits such as embryonic developmental rate (Robison et al. 2001; Sundin et al. 2005) and the timing of seasonal ovulation of eggs in females or female spawn timing (Sakamoto et al. 1999; Leder et al. 2006) have been investigated. The data from the Sundin et al. (2005) study further suggested that there also could be a link between QTL regions regulating developmental rate and EM in rainbow trout, given that they observed an association between the alleles coupled to faster developmental rate on RT-8 and EM in a small sample of full-sibs derived from the same experimental families. This chromosomal region is of particular interest as it was first identified to carry genes that were associated with major spawn timing differences in female rainbow trout and does in fact constitute a major QTL region associated with spawn timing, explaining between 20% and ~50% of the within-family variance observed for the trait (Sakamoto et al. 1999; Leder et al. 2006). These initial findings strongly suggest that there are QTL regions within salmonids, and potentially other vertebrate species, that either have pleiotropic influences on life-history timing events or that may represent closely linked syntenic clusters of genes regulating these multiple traits.

This study was aimed at identifying QTL locations underlying the onset of precocious maturation in rainbow trout and is the first to investigate the incidence of sex-specific QTL for EM in salmonids. Sex-specific QTL, especially weak or moderate ones, are more easily located with a sex-specific data set, as separate analyses by progeny sex remove the confounding factors associated with genotype by sex interactions (MacKay 2001). We were also interested in ascertaining whether developmental and maturation rates would be coupled at the genetic level. We reconfirm the existence of a strong QTL region on RT-8 regulating maturation timing in rainbow trout and show that genes within RT-8 direct EM in both males and females. Additional evidence for coupled developmental rate and EM QTL regions within this species is also provided. We also identify a number of candidate genes that either fall within the genomic QTL regions or are potentially located within these chromosomal segments, providing a paradigm for further investigations into this complex vertebrate life-history trait.

## Materials and Methods

### Fish Pedigree and Rearing History

Six half-sib families of rainbow trout (third generation of known pedigree originally derived from two pure commercial hatchery strains, Spring Valley (SV), a fast growing strain with a higher incidence of precociously maturing males, and Rainbow Springs (RS), a slower growing strain with a lower incidence of precociously maturing males (Martyniuk et al. 2003) were used in this study. All the parents were derived from crosses made in the fall of 1996. The families were created from ova and milt collected on October 7, 1999 (W0 = day of fertilization or day 0) and were reared at the Alma Aquaculture Research Station (Alma, Ontario, Canada) under similar conditions including a natural photoperiod regime and a constant 11°C water temperature for the duration of the experiment. The crosses were made using three hybrid inter-strain males derived from a single family crossed to a pure-strain SV female and a hybrid inter-strain female to produce the six diallel half-sib families (Table 1). All three brothers used in the diallel cross were precociously maturing males, with males 96-7-C2 and 96-7-C4 exhibiting the first signs of testes maturation (i.e., milt extrusion) on September 14, 1998 and male 96-7-C1 on October 5, 1998. Fish were fed a ration corresponding to the thermal growth coefficients devised for rainbow trout (approximately 2–3% of body weight daily) (Alanara et al. 2001). This ration was adjusted bimonthly according to the mean biomass of fish per tank and was reduced as the fish grew and reached maturation.

**Table 1:** Family pedigree used to detect QTL for growth and maturation timing in rainbow trout from two commercial strains: Spring Valley (SV) and Rainbow Springs (RS)

G_2_ sire	G_2_ dam	G_3_ family
96-7-C1^a^ (SV/RS)^b^	96-1-B5 (SV)	99-1 (*N* = 46)^c^
	96-7-B11 (SV/RS)	99-5 (*N* = 48)
96-7-C2 (SV/RS)	96-1-B5 (SV)	99-2 (*N* = 47)
	96-7-B11 (SV/RS)	99-6 (*N* = 47)
96-7-C4 (SV/RS)	96-1-B5 (SV)	99-4 (*N* = 50)
	96-7-B11 (SV/RS)	99-8 (*N* = 49)

^a^The parental designations XX-Y-Z#, where XX refers to the year that the cross was made, Y refers to the diallel lot, Z refers to the family designation in the 2 × 2 diallel lot, and # refers to the individual within the family

^b^Designations in brackets indicate the parental origin of the fish (i.e., pure-strain SV or SV/RS hybrid)

^c^Numbers in brackets indicate number of progeny used for each family

On day 257 (=W257), the fish were weighed and tagged with passive integrated transponders (PIT). Based on these weights, 25 of the largest and 25 of the smallest fish from each family were selected for the remainder of the study. These 300 fish (50 from each of the six families) were then reared together in a single 2-m diameter tank at the Alma hatchery until each family was randomly split into two equally sized groups and placed into one of two 2-m diameter tanks where they were then reared. On W372, the fish were weighed and transferred to a large (20 × 1.5 m) elongated indoor raceway where they were housed for the remainder of the experiment. Not all 50 fish reared from each family survived and 287 individuals out of an initial 300 were used for the final analysis.

### Phenotypic Data Collection

Maturation status was assessed from W608 onward and early-maturing individuals were identified on W735, W790, and W839. All fish recorded as mature during these dates (i.e., up to 2.3 years of age) were considered to be precociously mature. Fish were anesthetized with either 2-phenoxyethanol or tricaine methanesulfonate and scanned for PIT tag identification as well as being weighed and measured before being returned to their holding tank. Maturation status was ascertained by squeezing the fish to check for the presence of milt or ova.

### Genetic Marker Analysis

Genomic DNA was extracted from either white muscle, liver, gill, or adipose fin using a standard phenol chloroform protocol (Taggart et al. 1992) and microsatellite markers were genotyped according to the procedures described in Moghadam et al. (2007a). One hundred loci across 29 linkage groups were used in this study (see Supplementary Tables 1 and 2; supplementary tables and figures are available at: http://www.uoguelph.ca/~rdanzman/appendices/ and online). Both microsatellite locus designations and accession numbers were used to designate a given locus, and results are presented for the salmonid locus designations if information on the marker primer set has been published. Otherwise, accession numbers are listed to facilitate easier searches to the National Center for Biotechnology Information database.

### Linkage Analysis

Goodness-of-fit *G*-tests were performed to determine if the segregation of markers from an individual parent corresponded to the expected 1:1 Mendelian ratio. The markers that deviated from Mendelian expectations were further checked for large numbers of uninformative or missing progeny. Pairwise linkage among pairs of microsatellite loci and chromosomal phase of each polymorphic marker were tested using the program LINKMFEX (available at: http://www.uoguelph.ca/~rdanzman/software). The nature of marker choice (genome scan) ensured that the location of all markers was known a priori (Sakamoto et al. 2000; Danzmann et al. 2005). We also selected markers based on the criteria that at least one marker would be genotyped from each known chromosome arm in the current linkage map. A logarithm of odds (LOD) threshold value of 3.0 was used to test for linkage between markers. Where markers were assigned to linkage groups other than those designated in previous studies, the critical LOD value was raised to 4.0. Linkage maps were constructed separately for each sex due to the large recombination differences that exist between males and females (Sakamoto et al. 2000; Danzmann et al. 2005).

### QTL Detection and Analysis

QTL analysis was performed separately by progeny sex for segregating maternal and paternal alleles as the number of maturing progeny differed significantly between the sexes (Table 2). Early maturation was treated as a binary trait, with sexual maturation at or before 2 years of age scored as ‘0’ and the absence of early sexual maturation as ‘1’. QTL analysis was done using the J/qtl graphical interface (http://www.jax.org/staff/churchill/labsite/software) available for R/qtl (Broman et al. 2003) (http://www.rqtl.org) by implementing the binary model to test 1,000 permutations of the genotypic vectors generated for each parent using EM likelihood. This approach allowed us to obtain estimates for genome-wide significance levels of segregating markers tested within individuals. Single QTL locations were identified (scanone) across all linkage groups and accepted at the (0.05) confidence level for genome-wide significance. We also tested the distribution of phenotypic effects within individual linkage groups for chromosome-wide significant effects. Generally, those instances where markers were identified as being significant when linkage group assemblages were tested separately could be predicted from the genome-wide significance analysis as linkage groups surpassing the 37% confidence level following the latter analysis (see Supplementary Fig. 2).

**Table 2:** 2 × 2 *χ* ^2^ test comparing the incidence of early maturation in male and female progeny in six families of rainbow trout

Family	Female		Male		*χ* ^2^	*P*-value
	Mature	Immature	Mature	Immature		
99-1	2	16	16	12	9.75	<0.001
99-2	5	20	15	7	11.11	<0.001
99-4	6	19	17	8	9.74	<0.001
99-5	5	22	8	13	2.52	0.110
99-6	2	25	16	4	26.42	<0.001
99-8	1	27	14	7	22.49	<0.001

The number of mature (up to and including W839) and immature progeny of each sex are given per family

To further assess the accuracy of the QTL locations identified, we conducted a single-point analysis of all markers genotyped whereby genotypic scores were compared against the sexual status of each fish using a 2 × 2 contingency *χ* ^2^ test to determine the probability of the association of each parental allele with early maturation. Those markers which displayed a significant association with the presence or absence of sexual maturation were tested further using a contingency *χ* ^2^ bootstrapping algorithm using 10,000 bootstrapping replicates to increase the accuracy of the confidence level (Danzmann and Ihssen 1995).

Due to the ‘tail-end of distribution sampling’, subsequent analyses may be biased in favor of detecting QTL regions and may also inflate the associated QTL effects detected (Darvasi and Soller 1994). However, this method is recognized as an efficient means to increase the power of QTL detection as a consequence of increasing the phenotypic variance analyzed. Additionally, the intrinsic genetic background differences in the progeny derived from backcrosses and F_2_ intercrosses derived from two strains that differ substantially in the propensity for EM should lead to an increased probability of detecting genomic regions influencing EM. As a consequence, however, these types of analyses also increase the risk of inflating type I error levels. Therefore, we considered the most reliable QTL associations detected in this study are those for which both significant effects were detected as well as effects being seen in at least two of the five parents using the single-point analysis method.

To correct for the likely increase in type I errors that would result from the increased number of significance tests performed with the single-point analysis, two significance test corrections were applied. The first correction method was a modified Bonferroni correction utilizing genetic information across all test parents where the alpha level for significance was adjusted to: *ά* = 0.05 / *k* − 1; where *k* = the number of non-zero map positions genotyped in each mapping parent. In salmonids, males have substantially reduced recombination levels compared to females (Sakamoto et al. 2000; Gharbi et al. 2006), and thus two or more independent markers genotyped in a female parent could possess zero recombination in the male parent. In instances where markers shared identical map positions, the marker with the greatest genotypic information was used in the analysis. A modified *ά* was adopted as: *ά*″ = *ά* / *n*; where *n* = the number of parents observed to possess a putative QTL effect at that chromosomal location following the Monte Carlo bootstrapping procedure.

A second method involved using the Bayesian derived *q*-value False Discovery Rate (FDR) test using the R-language executable bootstrapping program called Qvalue. This program is available at: http://faculty.washington.edu/~jstorey/qvalue/ (Storey et al. 2004). As with the modified Bonferroni procedure, all the non-zero recombination *P*-values obtained for each marker within any given parent were tested. Also, similar to the above procedure, the results for male and female progeny were analyzed separately. Results obtained from the genome-wide EM likelihood searches were considered the most reliable for the reasons stated previously and comparisons of the QTL locations identified with this method to single-point analysis markers passing either the modified Bonferroni test or the Bayesian FDR test were largely congruent.

### Pedigree Analysis

To assess the origin of the alleles contributing to precocious maturation, we traced the inheritance of the alleles in the three full-sib brothers to their parental origins and related this information to the association of alleles with either late or early maturation within significant QTL marker regions. The 97-7-C family was derived from a cross between a pure SV female (93-32-3) and pure RS male (94-37-8). We were particularly interested in determining whether alleles transmitted from the SV strain would be more likely to be associated with the incidence of early maturation.

## Results

### Phenotypic Analysis

Sex-specific maturation rates were not observed to differ in the genetic background of either female, although female progeny maturation levels were marginally (*P* = 0.082) elevated in 96-1-B5 (Table 2). No differences in female progeny maturation proportions were observed across the three males tested, while male 96-7-C1 was observed to sire a significantly lower percentage of early-maturing male progeny compared to male 96-7-C2 (*P* = 0.02) and marginally lower percentage compared to male 96-7-C4 (*P* = 0.076). No differences in either male or female progeny maturation proportions were observed in the background of 96-1-B5 when crossed to all three males, and thus genotypic data were pooled for analysis within this female. Similarly, no differences in female progeny maturation schedules were observed for 96-7-B11 when crossed to all three males. However, male progeny maturation levels were observed to be significantly reduced (*P* = 0.025) in the mating with male 96-7-C1 (Table 2), and therefore EM QTL effects based on male offspring from this female were examined using family 5 data alone vs. families 6 and 8 pooled genotypic data to account for the observed interaction effects. The Bonferroni significance level for this ‘split’ analysis was set at: 0.05 / (128 − 1). The proportion of females with early maturation from family 4 was greater than that for family 8, but this result was not statistically significant. Since no significant differences in female or male maturation schedules were observed in the offspring of the three male parents when mated to either female, genotypic data were pooled for the statistical analysis for EM QTL within each male.

### QTL Associated with Early Maturation

Information on the number of markers genotyped within each rainbow trout linkage group is provided in the supplementary data (Supplementary Table 2). Linkage analysis confirmed the linkage associations and marker orders of the 100 markers used based on previous work (Danzmann et al. 2005).

Point analyses of the single-marker positions suggested the presence of a greater number of possible QTL locations (all data not shown) within the genome of rainbow trout compared to the binary EM likelihood tests, and thus the latter method is more conservative. Heterogeneous marker distributions (i.e., variable missing genotypes among markers) could result in significant marker assignments for one marker while an adjoining marker with a different distribution is not detected as being significant.

**Fig. 1:**
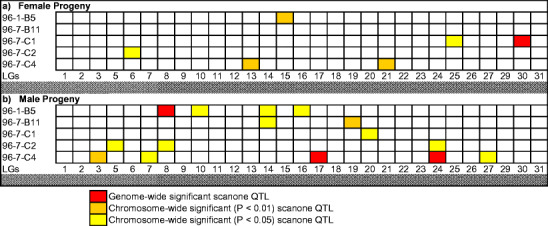
Significant genome-wide and chromosome-wide suggestive linkage group locations across the rainbow trout genome. Markers associated with the various QTL regions within each linkage group are listed in Tables 4 and 5

Genome-wide significant locations were located on RT-30 in female progeny (Table 3) and on RT-8, -17, and -24 in male progeny (Table 4) (see Fig. 1 for a summary of all genome-wide and chromosome-wide significant locations and Supplementary Fig. 1 for R/qtl plots across linkage groups). Generally, there was good congruence between the correction procedures for multiple testing from the single-point analyses and the R/qtl binary analysis. The FDR test appeared to be somewhat less conservative than the modified Bonferroni procedure. Six significant QTL were detected with the former method, while five were detected with the Bonferroni procedure. Four significant QTL were detected with both methods (see Tables 3 and 4). Significant EM QTL were detected on two linkage groups (RT-15 and -30) in female progeny. In the male progeny, significant QTL were localized to RT-3, -8, -17, and -24. When the male parent interaction effects for male progeny maturation timing in the genomic background of female 96-7-B11 were considered, the QTL localized to RT-19 was observed to be significant in the family backgrounds of males 96-7-C2 and -C4 using both single-point correction methods. The additive effects, although in a similar direction, were of much weaker influence in the male 96-7-C1 background (data not shown).

**Table 3:** Early maturation QTL detected in female progeny

LG	Parent (gender)	Marker	N1	N2	*χ* ^2^ (1 *df*)	*P*-value^a^	0.05^b^	0.01^b^	0.05^c^	0.01^c^
6	96-7-C2 (M)	OMM1082	23	18	5.991	0.0089	X			
		OMM1205	27	24	4.548	0.0341				
		OMM1302	28	20	5.853	0.0128				
		OMM1359	26	20	3.781	0.0499				
13	96-7-C4 (M)	OMM1321	36	19	5.679	0.0121		X		
		OmyRGT14TUF	35	19	5.862	0.0135				
15	96-1-B5 (F)	OMM1051	26	38	10.450	0.0013^3^		X		
		OMM1166	34	35	6.674	0.0088				
21	96-7-C4 (M)	OMM1059	36	19	8.894	0.0016		X		
25	96-7-C1 (M)	OMM1193	17	12	5.340	0.0148	X			
30	96-7-C1 (M)	BX079862	17	23	4.816	0.0207				
		OMM1186	20	19	8.103	0.0015^1^				X

N1 and N2 represent the number of progeny with each allele segregating at the given locus. *χ* ^2^ values and the corresponding bootstrapping *P*-values are listed. Parent 96-1-B5 is a female (F) and 96-7-C1, -C2, and -C4 are male (M)

^a^Markers that remain significant following a Bonferroni correction method, the Bayesian correction method, or with both methods are indicated with a 2, 3, or 1 superscript, respectively

^b^Specific chromosome locations associated with the maximal QTL location for a binary trait analysis in R/qtl demonstrating chromosome-wide significance

^c^Specific chromosome locations associated with the maximal QTL location for a binary trait analysis in R/qtl demonstrating genome-wide significance

**Table 4:** Early maturation QTL detected in male progeny

LG	Parent (gender)	Marker	N1	N2	*χ* ^2^ (1 *df*)	*P*-value^a^	0.05^b^	0.01^b^	0.05^c^	0.01^c^
3	96-7-C4 (M)	BHMS206	19	21	4.606	0.0331				
		BX317661/i	22	20	4.772	0.0300				
		OMM1297	20	16	9.506	0.0012^3^		X		
5	96-7-C2 (M)	BX318599	28	10	4.211	0.0277	X			
7	96-7-C4 (M)	OMM1087	19	12	5.128	0.0234	X			
		OMM1305	14	26	5.358	0.0190	X			
8	96-1-B5 (F)	CA060381	30	30	5.710	0.0163				
		Omi134TUF	36	32	4.766	0.0241				
		OMM1304	26	43	9.937	0.0015^2^			X	
		OmyFGT12TUF	32	38	4.917	0.0279				
		One112ADFG	35	33	5.567	0.0177				
		One114ADFG	35	25	4.238	0.0369				
	96-7-C2 (M)	Ots532NWFSC	20	21	5.633	0.0170	X			
10	96-1-B5 (F)	Omy1225UW	22	18	7.930	0.0049	X			
14	96-1-B5 (F)	OMM1657	19	29	5.289	0.0200	X			
14	96-7-B11 (F)	OMM1134	19	30	5.222	0.0230	X			
17	96-7-C4 (M)	BX305863	21	22	14.442	0.0000^1^				X
19	96-7-B11 (F)	BX298853	33	25	7.600	0.0050^1^		X		
		OMM1025	33	27	6.159	0.0110				
		Omy103INRA	28	29	3.996	0.0490				
20	96-7-C1 (M)	OMM1257	24	22	5.575	0.0177	X			
24	96-7-C2 (M)	OMM1105	21	19	5.229	0.0194	X			
		Omy27INRA	23	19	4.545	0.0301				
	96-7-C4 (M)	BHMS377	15	25	4.478	0.0270				
		OMM1105	26	12	9.993	0.0010^1^				
		OMM1320	10	18	5.534	0.0170			X	
		OMM1322	14	27	10.420	0.0000^1^				
		Omy4DIAS	15	28	7.900	0.0040				
27	96-7-C4 (M)	BX073867	17	24	5.330	0.0210	X			
		CA374878	19	17	4.887	0.0250	X			

N1 and N2 represent the number of progeny with each allele segregating at the given locus. *χ* ^2^ values and the corresponding bootstrapping *P*-values are listed. Parents 96-1-B5 and 96-7-B11 are female (F) and 96-7-C1, -C2, and -C4 are male (M)

^a^Markers that remain significant following a Bonferroni correction method, the Bayesian correction method, or with both methods are indicated with a 2, 3, or 1 superscript, respectively

^b^Specific chromosome locations associated with the maximal QTL location for a binary trait analysis in R/qtl demonstrating chromosome-wide significance

^c^Specific chromosome locations associated with the maximal QTL location for a binary trait analysis in R/qtl demonstrating genome-wide significance

While the genetic regions identified on RT-3, -15, and -19 were not identified as possessing genome-wide significance using the scanone method in R/qtl, these three linkage groups were identified as having highly significant (*P* < 0.01) chromosome-wide significance levels along with markers on RT-13 and RT-21 (see Tables 3 and 4). The only major discrepancy between the single-point and binary analysis in R/qtl was in relation to a significant chromosome-wide association detected for marker OMM1015 on RT-26 which was not supported with the single-point analysis.

### Pedigree Analysis

Within four of the significant EM QTL locations detected within the three 96-7-C brothers, the allele transmitted via their RS father was associated with EM almost exclusively in their sons. However, for the significant EM QTL detected on RT-30, observed in female progeny, the allele origin associated with EM was from the SV mother (Table 5; Supplementary Fig. 2). Interestingly, with one of the markers on RT-24 (i.e., OMM1320), the QTL effect in sons derived from sires 96-7-C4 and 96-7-C2 was associated with their grand-paternal allele, while in 96-7-C1, this same allele was associated with EM in his daughters. For one of the markers, Ots532NWFSC on RT-8, an EM QTL effect was observed in the male progeny of 96-7-C2 associated with a grand-maternally transmitted allele, while in the male progeny derived from 96-7-C4, a grand-paternally transmitted allele at BHMS415 contributed to the EM QTL (Table 5).

**Table 5:** Identification of maternally (SV origin) or paternally (RS origin) EM QTL alleles in the male and female progeny of three full-sib brothers from the 96-7-C inter-strain hybrid family

LG	Marker	Female parent	Male parent	96-7-C1		96-7-C2		96-7-C4		Faster^a^ EM allele
				♀ allele	♂ allele	♀ allele	♂ allele	♀ allele	♂ allele	
RT-3	BHMS206	132/162	144/152	132	152	162	152	162	*144*♂	*144*
RT-3	BX317661	205/209	191/197	209	197	205	197	205	*191*♂	*191*
RT-3	OMM1297	264/278	264/274	264	264	264	264	264	*274*♂	*274*
RT-8	Ots532NWFSC	180/180	180/198	**180**	198	*180*♂	198	180-?	198	*180*
RT-8	BHMS415	129/129	129/143	129	143-?	129	143-?	129	*143*♂	*129 + 143*
RT-8	One112ADFG	149/149	113/179	149	179	149	179	149	179	
RT-8	OmyFGT12TUF	168/182	138/186	182	138	182	138	182	138	
RT-8	CA060381	160/214	168/220	214	168	160	168	214	168	
RT-8	OMM1009	245/245	245/251	245	251	245	251	245	251	
RT-8	One114ADFG	220/224	198/220	220	198	220	220	220	198	
RT-17	BX305863	255/223	215/229	223	229	223	**215**	255	*215*♂	*215*
RT-24	BHMS377	128/132	138/138	128	138-?	132	*138*♂	132	*138*♂	*138*
RT-24	Omy4DIAS	134/134	126/160	134	**160**	134	*160*♂	134	*160*♂	*160*
RT-24	OMM1105	125/183	159/183	125	**159**	125	*159*♂	183	*159*♂	*159*
RT-24	OMM1320	116/116	124/124	116	*124*♀	116	*124*♂	116	*124*♂	*124*
RT-24	OMM1322	199/184	191/231	184	191-?	184	*191*♂	184	*191*♂	*191*
RT-30	BX079862	151/165	155/157	*151*♀	155	151-?	157	165	155	*151*
RT-30	OMM1186	200/200	204/208	*200*♀	204	200-?	208	200-?	204	*200*

Alleles associated with earlier maturing offspring are indicated in italic font in the last column of the table. If the allele is coupled to a QTL effect in male progeny it is indicated with a ♂ symbol, while alleles coupled to the QTL region in daughters are indicated with a ♀ symbol. Alleles that are of similar descent and express an additive effect in the same direction as the EM QTL allele are indicated in bold font, while a reversal in the direction of the additive effect are indicated with a ‘-?’. Allele sizes in base pairs are given

^a^Allele associated with earlier maturation timing in the progeny

## Discussion

### Early Maturation QTL

The current data suggest that the onset of maturation in male and female rainbow trout might, in large part, be under separate genetic control. When considering both genome-wide and chromosome-wide significant effects, EM QTL in females were found on six (RT-6, -13, -15, -21, -25, and -30) and in males on twelve (RT-3, -5, -7, -8, -10, -14, -16, -17, -19, -20, -24, and -27) linkage groups, respectively. The most pronounced effect on female maturation schedules was observed on RT-30, while a strong QTL for male maturation schedules was detected on RT-17. Strong EM QTL for male progeny were also detected on RT-8 and -24. Similar findings have been reported in Arctic charr, where different chromosome regions appear to be associated with sex-specific differences in maturation timing (Moghadam et al. 2007a). Given the small number of parental genomes tested in this study, however, further data is required to more fully explore sex-specific QTL associations. Also, the fact that we observed significant associations following the single-point analyses (data not shown) within additional parents for a marker located on RT-8 (i.e., OmyFGT12TUF within female progeny descended from 96-1-B5 and 96-7-B11) as well as RT-24 (i.e., OMM1320 within female progeny descended from 96-7-C1) is suggestive that the EM QTL localized to these linkage groups may generally act in non-sex-specific fashion or generate associations that appear generic. For example, when analyzing the combined male and female progeny distributions within parents using sex as a co-variate with the binary model in R/qtl, only RT-17 was detected as possessing significant genome-wide significance (data not shown). The highly reduced, or reversed (i.e., RT-8), allelic associations in the opposite sex for the QTL localized to RT-8, -24, and -30 suggest that negative interactions with sex within or between QTL regions may occur. For the inconsistent allelic associations observed in RT-8, the data from female 96-1-B5 suggest that two interacting QTL (see Supplementary Figs. 1 and 3) regions occur in this linkage group.

**Fig. 2:**
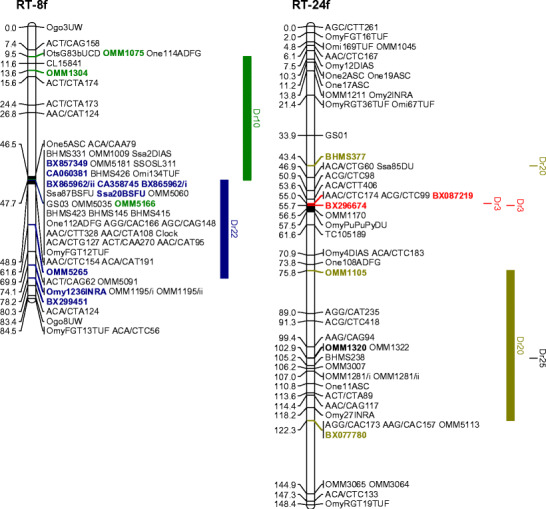

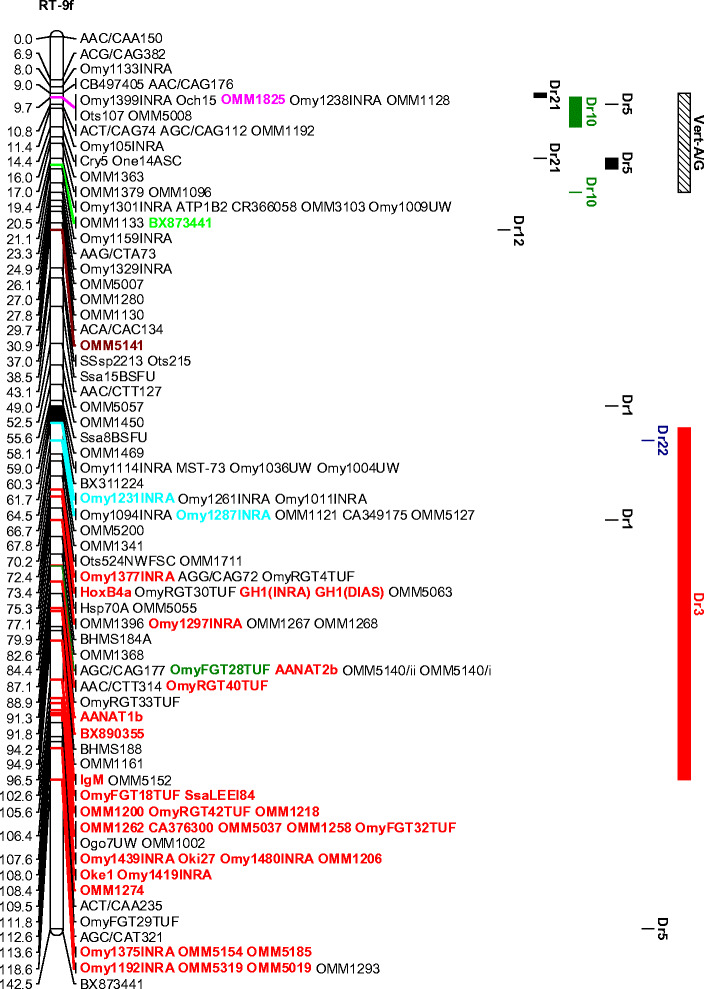
**a** Composite female-based linkage map of RT-8 and RT-24 in rainbow trout, using updated mapping data from the Danzmann et al. (2005) mapping panels. Inferred homologies to the zebrafish (*Danio rerio*) linkage groups based upon BLASTN affinities are *shown to the right* according to the following colors: Dr3 (*red*), Dr10 (*green*), Dr20 (*dark yellow*), and Dr22 (*blue*). **b** Composite female-based linkage map of RT-9 in rainbow trout, using updated mapping data from the Danzmann et al. (2005) mapping panels and the gynogenetic haploid mapping panel of Guyomard et al. (2006). Identified putative homeologies within this linkage group are based upon the following marker colors: RT-8/9 (*pink*), RT-2/9 (*red*), RT-9/20 (*bright green*), RT-9/24 (*turquoise*), RT-9/13 (*sea green*), and RT-9/22 (*plum*). Inferred homologies to zebrafish (*Danio rerio*) linkage groups based upon BLASTN affinities are shown to the right of RT-9. Centromeres are depicted as *solid black segments* within each linkage group and linkage groups are oriented with the short (p) arm towards the top of each figure. Orientations are based upon fluorescence in situ hybridization results from marker probes (Phillips et al. 2006)

The EM QTL marked by OmyFGT12TUF, Ots532NWFSC, and OMM1304 on RT-8 and OMM1320, OMM1322, and OMM1105 on RT-24, respectively, were all localized to regions of these linkage groups that could be localized with additional marker information from other mapping panels. In the case of linkage group RT-24, markers OMM1320 and OMM1322 map very close to one another and have zero recombination in some mapping panels (Danzmann et al. 2005), while OMM1105 is located even more proximal to the centromeric region. All these markers map to the long arm of RT-24 (i.e., RT-24q) as determined from the in situ hybridization results from selected markers (R. Phillips, personal communication) (see Fig. 2). The localization of markers onto the chromosome arms of RT-8 is somewhat problematic given the unusual lack of recombination noted in both males and females for this chromosome group (Danzmann et al. 2005). Many markers appear to localize centrally within the linkage group with more informative meioses restricted to more telomeric regions. Nonetheless, in this study, we were able to ascertain that the strongest QTL position (in male progeny) appears to localize to the 8p arm (marked by OMM1304 in 96-1-B5) (see Fig. 2), although marker regions located within the central cluster of this linkage group may also contribute to the phenotypic effects, as mentioned in the previous paragraph. In this regard, it is also of interest to note that regions of RT-8 and RT-24 may show ancestral homeology in rainbow trout, given the recent finding that duplicated copies of glutamine synthetase map to these two linkage groups (Gharbi et al. 2007). Guyomard et al. (2006) have also reported homeology between the telomeric region of the short arm of RT-9 and RT-8 (i.e., OMM1825) and a pair of centromeric markers on RT-9 and the centromeric region of RT-24 (see Fig. 2). Aside from the report of single duplicated markers suggesting homeology between RT-1/8 (Phillips et al. 2006), RT-8/20 (Guyomard et al. 2006), and RT-23/24 (Danzmann et al. 2005), the homeologous affinities for the fourth (i.e., RT-24) and fifth (i.e., RT-8) largest chromosomes in the rainbow trout genome (Phillips et al. 2006) are largely unknown.

The RT-9p arm is of particular interest given that homology to several markers on zebrafish chromosomes Dr-5, Dr-10, and Dr-21 have been identified within this arm (unpublished data). This suite of linkage groups is derived from interchanges among the ancestral vertebrate A/G proto-linkage groups (Jaillon et al. 2004; Woods et al. 2005), and therefore affinities between RT-8/9 may reflect more ancient 3R affinities to the ancestral vertebrate–A/G grouping as well. This is also supported by the observation that several markers on RT-20 share homology to Dr-10, as do centromeric and telomeric regions on RT-8. Conversely, centromeric regions on RT-24 share homology to Dr-3, as do a very large number of markers on RT-9q (see Fig. 2). If RT-8/24 do indeed share some degree of homeology, then an ancestral region controlling EM may have been conserved, explaining the EM QTL in male and female progeny detected on RT-8 and -24. However, based upon the comparative data with the zebrafish genome, this scenario cannot currently be supported without more extensive fine-mapping information within the regions surrounding the QTL.

The linkage groups mentioned in the previous paragraph are also of interest in an evolutionary context, given that they collectively represent multiple putative homeologous groupings within the rainbow trout genome arising from the more recent whole genome duplication event in salmonid fishes compared to other teleost fishes (Allendorf and Thorgaard 1984). When considering the complete dataset in which significant genome-wide and chromosome-wide scanone QTL are considered, potentially duplicated EM QTL effects could be localized within the homeologs or homeologous regions of RT-3/25, RT-6/30, RT-7/15, RT-7/19, RT-8/24, and RT-14/20.

Metacentric chromosomes are expected to possess at least two whole-arm homeologies while acrocentrics should possess a single affinity. Linkage groups 25 and 30 represent acrocentric chromosomes within rainbow trout (Phillips et al. 2006); however, in the mapping panels examined by Danzmann et al.( 2005), it appeared that RT-25 represented a metacentric fusion between RT-4 and RT-25. Such inter- and intra-population polymorphisms in chromosome number are known to exist in rainbow trout (Thorgaard 1983). In our study, we cannot unequivocally relate the homeology in RT-25 to its respective position on RT-3, given the fact that the QTL were detected in male parents and that we did not karyotype the male parents in the study to ascertain if they possess 58 or 60 chromosomes. Similarly, for the EM QTL on RT-6, we cannot unequivocally assign the QTL location to either chromosome arm for this metacentric linkage group given that the QTL effect was localized in male fish; however, the location of OMM1082 does fall onto RT-6q which is the arm sharing homeology with RT-30.

Both of the linkage group arm markers in male 96-7-C4 for RT-7 exhibited chromosome-wide significance, and thus we cannot unequivocally confirm the homeology of RT-7p/15p. Furthermore, the localization of the EM QTL in female 96-1-B5 was to a marker close to the centromeric region of RT-15, which further complicates distinct arm assignments. Rearrangements within the centromeric region on RT-7 may also have occurred as the region housing OMM1087 still shares synteny with genomic regions of zebrafish Dr-4, as does almost the entire arm of RT-7p (data not shown). Unfortunately, we did not genotype any markers on the more distal sections of RT-7q that share homology to Dr-24. The relative strength of QTL associations for markers in this distal region would have supported or refuted the putative and the possible homeology for QTL effects between RT-7p/15p.

Centromeric regions of both RT-7 and RT-19 share some degree of homology/homeology to one another as evidenced from the mapping of duplicated copies of the *PACAP* gene to these regions (Moghadam et al. 2007b). Therefore, a putative duplicated homeologous QTL region could exist between these two linkage group regions given the fact that OMM1025 is located on the centromere region of RT-19 and the QTL effect on RT-7 within male 96-7-C4 is compatible with assignment to either linkage group arm. The localization of putative homeologous affinities between RT-14/20 cannot be fully ascertained from the current study as the chromosome-wide EM QTL localized to RT-14p cannot be related to the RT-20 marker (i.e., OMM1257). This is due to the fact that the most complete marker information in this region is derived from male mapping parents (i.e., lacks recombination information). However, the fact that OMM1257 remained unlinked to OMM1657 in the female 96-1-B5 (see Supplementary Table 2) suggests that OMM1257 may be localized to RT-20q (i.e., the arm opposite to the one sharing synteny with RT-14p). This would then suggest that the RT-14/20 QTL detected in this study do not represent homeologous affinities.

### Associations with Growth

We also performed a QTL analysis based upon growth and condition factor changes in these same fish throughout their early juvenile growth phases using either five or six independent samplings of the fish growth within each family starting at day 257 of development. Growth is known to be physiologically coupled to endogenous triggers initiating sexual maturation in salmonid fishes (Thorpe et al. 1983; Rowe et al. 1991), and thus it was not surprising to observe either body weight or condition factor QTL co-localized to the same chromosomal regions as the EM QTL (data not shown; unpublished). Interestingly, however, while the same parent would most often exhibit a growth-related QTL coupled with an EM QTL, this was not true for the QTL associated with EM in female progeny, for parents 96-1-B5, 96-7-B11, and 96-7-C1, within the homeologs RT-6/30. Although growth QTL were located on these chromosome arms in at least one other parent, the lack of a direct coupling of growth and EM QTL in the same individual suggests that these traits may be more independently regulated within these ancestrally duplicated chromosomes with respect to female maturation timing schedules.

### Pedigree Analysis

The observation that chromosomal regions derived from a strain background exhibiting lower levels of precocious maturation may possess genetic regions contributing to a higher incidence of EM indicates that selection background may not necessarily be a good predictor of performance expression in hybrid backgrounds. However, this association was not entirely unexpected given the fact that the sire for the 96-7-C brothers was also a precociously maturing male in the autumn of 1995. For the majority of the allelic effects examined in the three 96-7-C males, when a significant effect was observed within one of the males and that same allele was transmitted to another brother, the allelic effect was in the same direction in their progeny. When exceptions or reversals in allelic effect were observed (e.g., BHMS377 and OMM1322 on RT-24 and BX079862 on RT-30), the reversal could be due to recombination between the QTL region and the marker (e.g., OMM1322) or contributions from alternate interacting alleles derived from the opposite parent (e.g., allele 128 bp at BHMS377 and allele 157 bp at BX079862 from male 94-37-8).

Reversals in allelic effect were observed across the three full-sib brothers for the EM QTL localized within RT-8. The allele associated with EM QTL in 96-7-C2 more often was associated with delayed maturation in the sons of 96-7-C4. Also, the suggestive EM QTL allele in 96-7-C4 more often was associated with delayed maturation in the sons of 96-7-C1 and -C2 (see Table 5). Given the fact, however, that significant EM QTL were also detected on RT-8 for both of the females used in the diallel crosses and that crossing over was also evident for the transmission of alleles to the three brothers from their mother (see CA060381 in Table 5), it is likely that both additive and epistatic interactions contributed to the observed QTL effects within not only RT-8 but the other QTL regions as well.

### Life-history Variation

The only previous study that attempted to identify EM QTL in rainbow trout identified three linkage groups (i.e., RT-13, -24, and -31) possessing QTL for this trait (Martyniuk et al. 2003). It should be pointed out, however, that these investigators only examined variation within seven linkage groups (i.e., RT-3, -9, -13, -21, -24, -27, and -31). Similarly, as reported in the “Introduction” section, Sundin et al. (2005) reported a potential association between developmental rate (DR), maturation timing, and variation within RT-8 as having an influence on this life-history trait. Our findings support the postulate by Sundin et al. (2005) that genomic regions within RT-8 appear to have an influence on these two life-history traits.

Robison et al. (2001) were the first to investigate QTL variation associated with developmental rate in rainbow trout and reported four major QTL associated with this trait. Subsequent research localized the strongest QTL for this trait (*tth-1*) to markers within RT-8. Additional work by Nichols et al. (2007) using a clonal line of rainbow trout identified major DR QTL on six linkage groups (i.e., RT-8, -9, -10, -14, and -24), including an unassigned cluster. The largest QTL effect was localized to RT-8. When considering both suggestive and significant findings, our data support the observation that DR QTL may be directly coupled to EM QTL, as all the linkage groups possessing DR QTL in the Nichols et al. (2007) study, with the exception of RT-9, were also associated with EM QTL in the current study. Furthermore, for three of these linkage groups (i.e., RT-8, -14, and -24), the effects were observed in multiple parents.

EM QTL regions also appear to share a high degree of similarity with differences in the onset of spawning time (SPT) in female rainbow trout, suggesting pleiotropy in the functions of the genes within these regions. It is also possible that multiple syntenic genes regulating these two life-history traits may occur within these chromosomal regions. Leder et al. (2006) reported significant SPT QTL for this trait on ten different linkage groups (i.e., RT-3, -7, -8, -11, -18, -19, -20, -22, -24, and -31), with the strongest effects localized to RT-3, RT-8, and -20. We have observed that significant EM and SPT QTL co-occur within the same linkage group regions on RT-3, -7, -8, -19, and -24.

The strong SPT QTL location on RT-22 reported by Leder et al. (2006) may be related to the significant EM QTL region on RT-17 given the fact that a pair of arms in these metacentric linkage groups are homeologous (Danzmann et al. 2005). Updated (unpublished) mapping data using the Leder et al. (2006) mapping panel has localized a moderate effect SPT QTL towards the distal end of RT-17 (i.e., associated with the EST marker BX073647 within the homeologous arm to RT-22) in the female parent. Interestingly, however, this effect was localized only within the female offspring in this family when they matured as 4-year-olds, while a region next to OMM1362 towards the telomeric end of RT-22p was associated with the strongest SPT QTL effect within these fish as 3-year-olds. Marker BX073647 and OMM1362 map approximately 2 cM apart. BX305863, the marker with the strongest EM QTL effect observed in this study, is linked with zero recombination to BX073647 on RT-17q in the Danzmann et al. (2005) mapping panels. These homeologs (i.e., RT-17q/22p) appear to share a large tract of synteny to zebrafish Dr-12 (see Supplementary Table 8).

### Candidate Genes

Two approaches were taken with respect to identifying the candidate genes underlying the expression of precocious maturation in the fishes studied. First, we used knowledge of the known genetic map positions of genes that could influence the expression of this trait. Genes mapping within the region of a QTL marker demonstrating at least a genome-wide or highly significant chromosome-wide QTL effect were considered putative candidates for the trait. For male-derived QTL positions, this largely resulted in an assignment to whole linkage groups, or chromosome arms, given the lack of recombination in male salmonids (Sakamoto et al. 2000). Our second approach was to infer potential candidate genes using a comparative genomics approach with the zebrafish genome. The map positions of markers within the composite female genetic maps for rainbow trout (Danzmann et al. 2005; Guyomard et al. 2006) were used as reference points to compare their sequences against the zebrafish genome (BLASTN near-exact matches and distant homologies using an *e* ≤ 10^−5^ cutoff for assignment) within the Ensembl database (http://www.ensembl.org). Zebrafish genes derived from a single chromosome that shared uninterrupted synteny (i.e., possessing two or more adjacent map positions) with markers from the rainbow trout map were considered potential candidates. For example, a large segment of Dr-22 appears to share homology to the central portion of RT-8, while the telomeric region of 8p in this linkage group shares synteny with segments of Dr-10. The centromeric regions of RT-24 appear syntenic to segments of Dr-3, while the region on RT-24q bearing the EM QTL appears syntenic to portions of Dr-20. A listing of potential candidates spanning each synteny block ±5 Mb from within the zebrafish genome (v7_September 2007 release) was obtained from the BIOMART database in Ensembl v.46 (see Supplementary Tables 3–12). Synteny blocks were detected for all the significant QTL locations on RT-3, -8, -15, -17, -19, and -24 but not for -30. On RT-30, markers with homology to Dr-12 and Dr-13 have been localized spanning the QTL peak at OMM1186 and on RT-17 spanning a region around BX305863. Zebrafish gonadotrophin II (*GtHII*), or more specifically the luteinizing hormone beta II (*lhb*) subunit, is located in the vicinity of Dr-13 homology and follicle stimulating hormone (or gonadotrophin I) receptor (*fshr*) occurs adjacent to the syntenic region on Dr-12, while the gene *GnRH-3A* has also been mapped to RT-30 within rainbow trout (Leder et al. 2004). Nichols et al. (2003) have previously mapped *lhb* to the centromeric region on RT-17 in rainbow trout, and thus this gene may account for the significant EM QTL located within this region. If gonadotrophin II expression primarily accounts for the RT-17 QTL, then it is unlikely that the influences of this gene will be restricted solely to male maturation schedules (detected in this study), given the fact that the *GtHII* and particularly the beta subunit is known to be a primary hormonal trigger in female ovulation rates (Devlin and Nagahama 2002).

The findings from this QTL study are particularly encouraging in that they appear to overlap key gene regions that have been linked to sexual development and steroidogenic ontogenies in salmonids and other fishes. For example, von Hofsten et al. (2002) reported significant changes in steroidogenic acute regulatory protein (*StAR*) and *Fushi Tarazu Factor-1* (NR5A2 regulatory receptor family) gene expression levels during sexual development in Arctic charr. Based upon BLASTN searches of their GenBank accessions to the zebrafish genome, these genes fall within the QTL regions we detected on RT-19 and RT-8, respectively (see Supplementary Tables 12 and 5, respectively). Also, the major gene regulating the onset of spermatagonial proliferation in Japanese eels (*Anguilla japonica*) (i.e., *eSRS21*) shows a high degree of homology to vertebrate anti-müllerian hormone (*amh*) (Miura et al. 2002), and this gene is located on Dr-22 within the region possessing putative homology to EM QTL on RT-8 (see Supplementary Table 5). Candidate gene locations identified from the comparative analyses with zebrafish genome as well as other model teleost genomes such as the medaka (*Oryzias latipes*) or actual mapped gene locations in the rainbow trout genome remain promising sources for future research into the physiological genomics of maturation onset in these fishes.

## Electronic supplementary material

Below is the link to the electronic supplementary material.

Supplementary Fig. 2 TAMRA-labelled DNA fragments generated for the various EM QTL markers detected in rainbow trout. Associations of marker to linkage groups are indicated by the *solid black bar* at the top of each set of markers. For each panel, the maternal parent (93-32-3) and paternal parent (94-37-8) of family 96-7-C are shown in the *left and rightmost lane*, respectively. One of the three full-sib brothers (i.e., 96-7-C4) is shown in the *middle lane*. Allele sizes (bp) for both of the parents and their three male offspring are given in Table 5. The smallest allele bp sizes are at the *bottom* of the panel and increase in size in ascending order within each figure. Panels are not represented to a uniform scale (GIF 126 kb)

Supplementary Fig. 3 Scantwo R/qtl plots from linkage group RT-8 depicting combined male- and female-specific QTL regions using sex as a co-variate. Epistasis LOD peaks are shown in the *upper left of the figure* according to the left-hand side of the LOD scale depicted. Additive LOD scores are shown in the *lower right* with the highest LOD peak (3.678) according to the right-hand side of the LOD scale depicted. The highest LOD score quadrant indicates that two contributing regions (OmyFGT12TUF and OMM1304) are likely present in the genome of this female (GIF 149 kb)
