# Supplementary figures and images for: Determination of Quantitative Trait Loci (QTL) for Early Maturation in Rainbow Trout (Oncorhynchus mykiss)
Source: Mar Biotechnol (NY). 2008 May 20;10(5):579–92. doi: 10.1007/s10126-008-9098-5 (PMC2516301; doi:10.1007/s10126-008-9098-5)

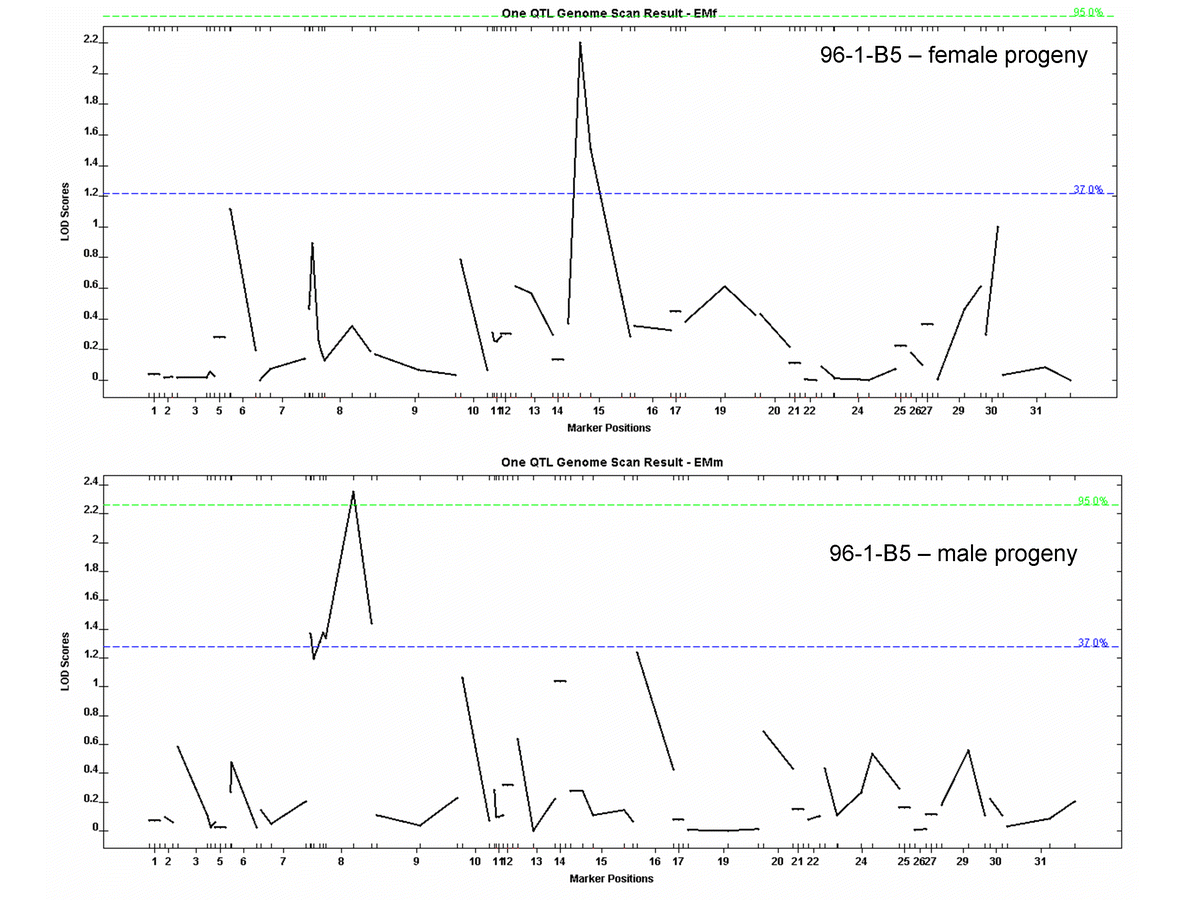

Supplement: Supplementary file 1 [file 10126_2008_9098_Fig1a_ESM.gif]

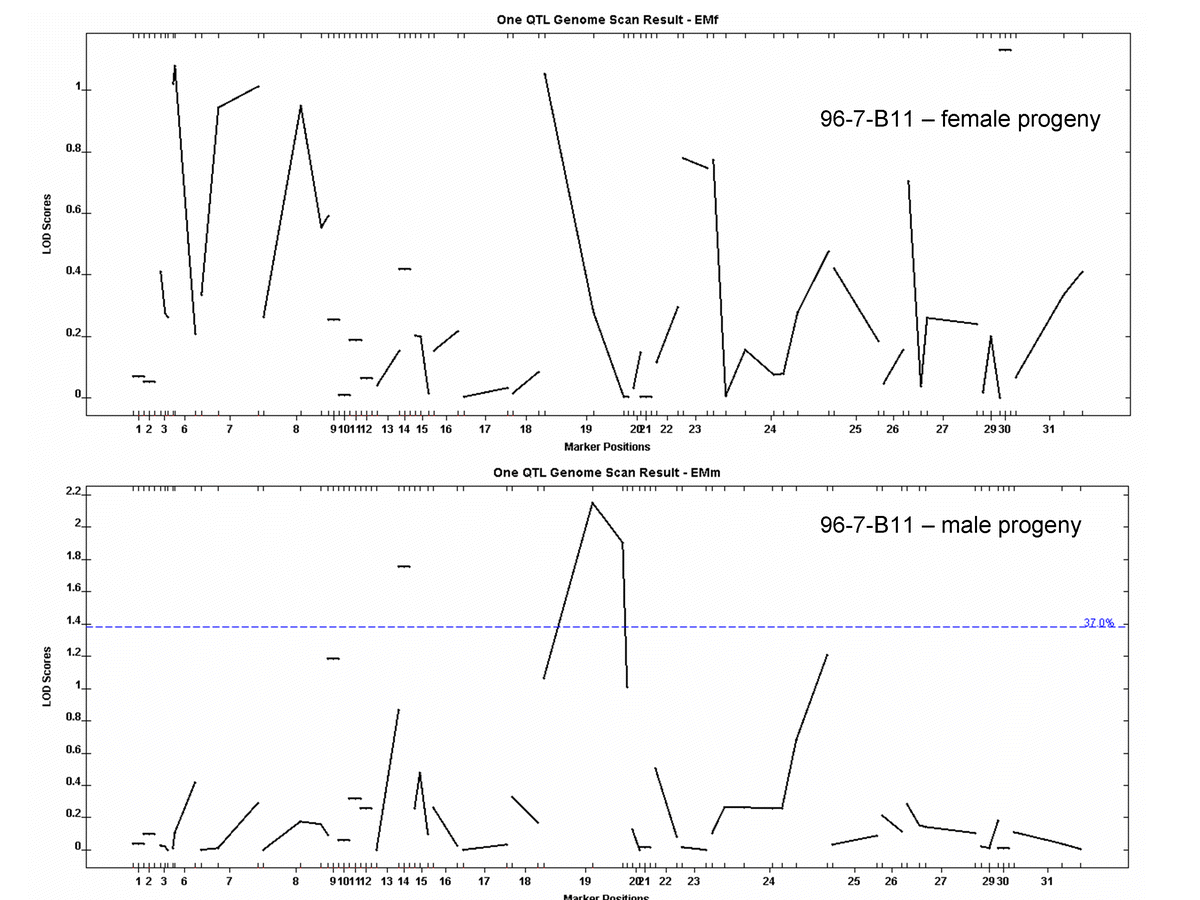

Supplement: Supplementary file 2 [file 10126_2008_9098_Fig1b_ESM.gif]

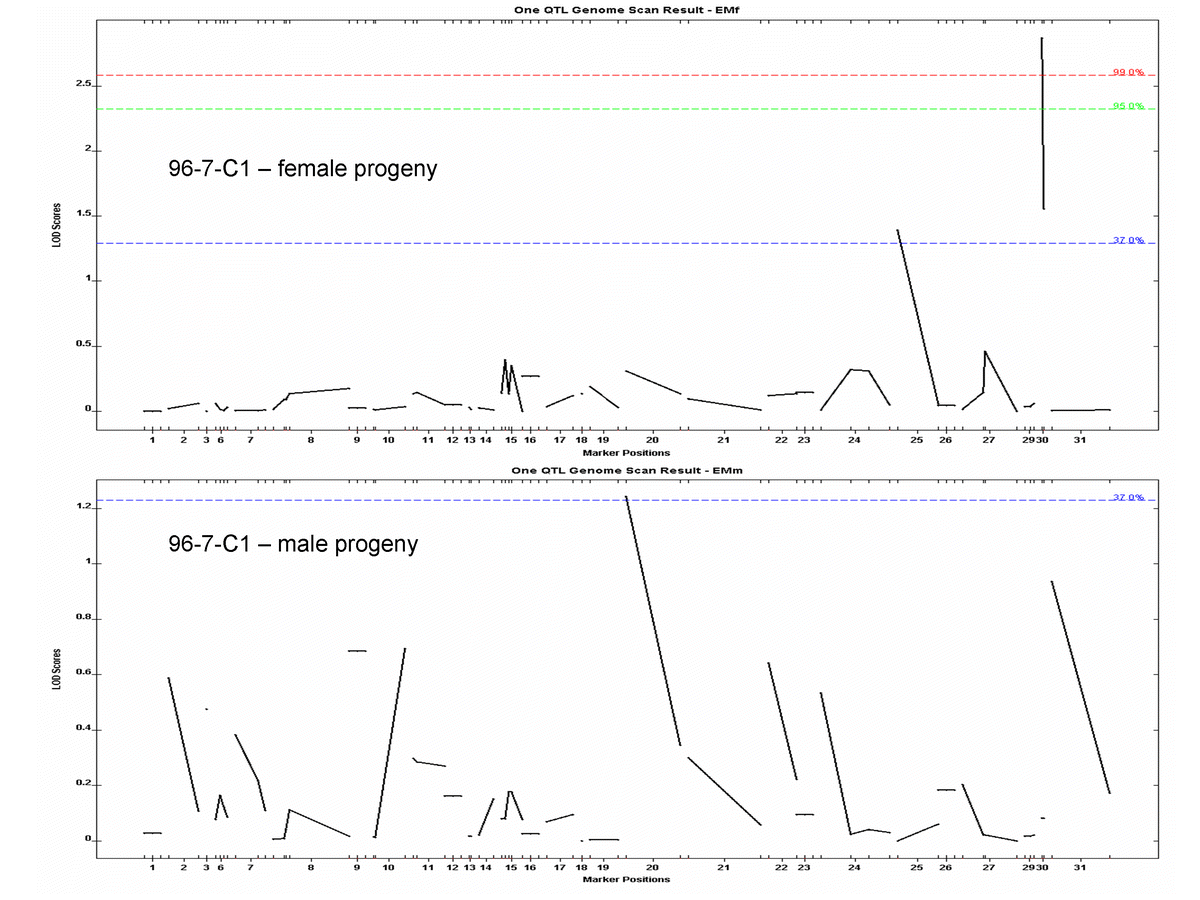

Supplement: Supplementary file 3 [file 10126_2008_9098_Fig1c_ESM.gif]

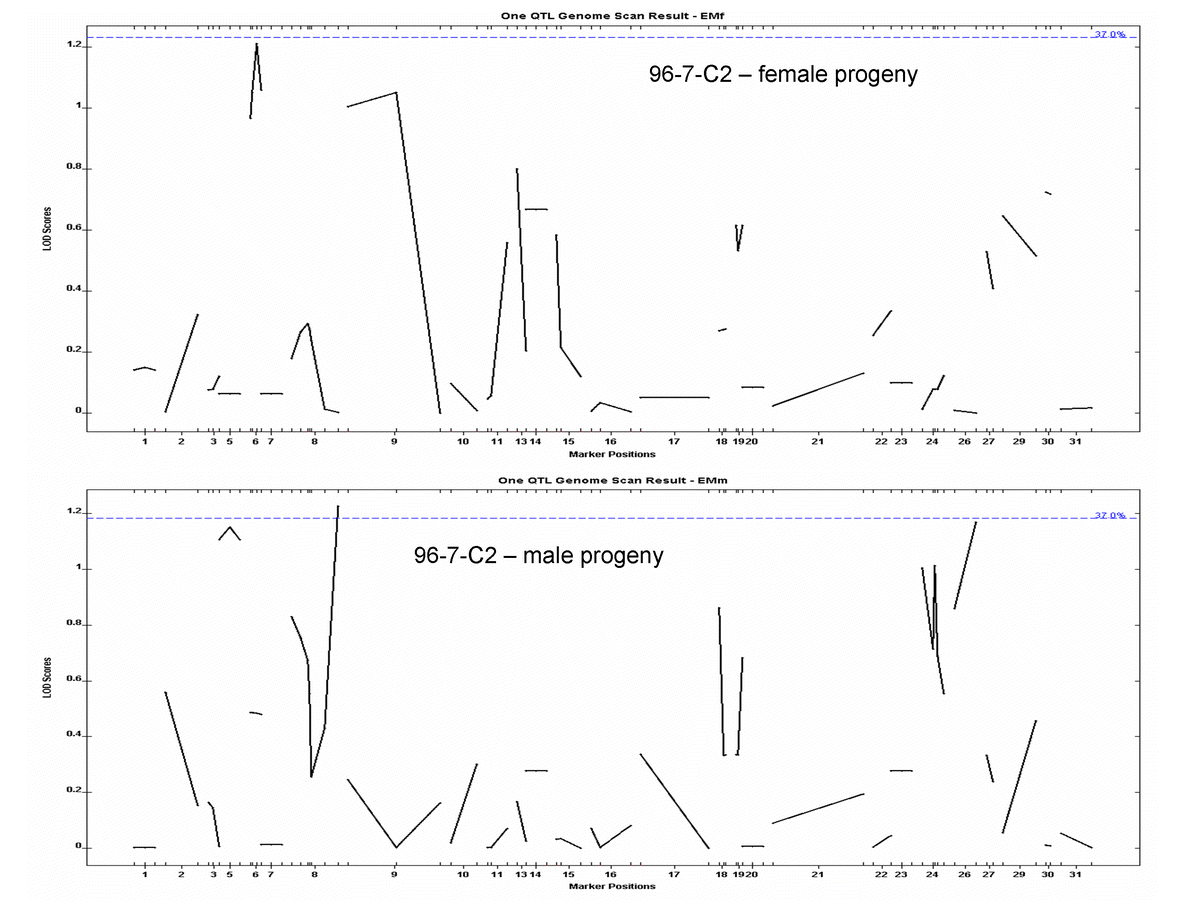

Supplement: Supplementary file 4 [file 10126_2008_9098_Fig1d_ESM.gif]

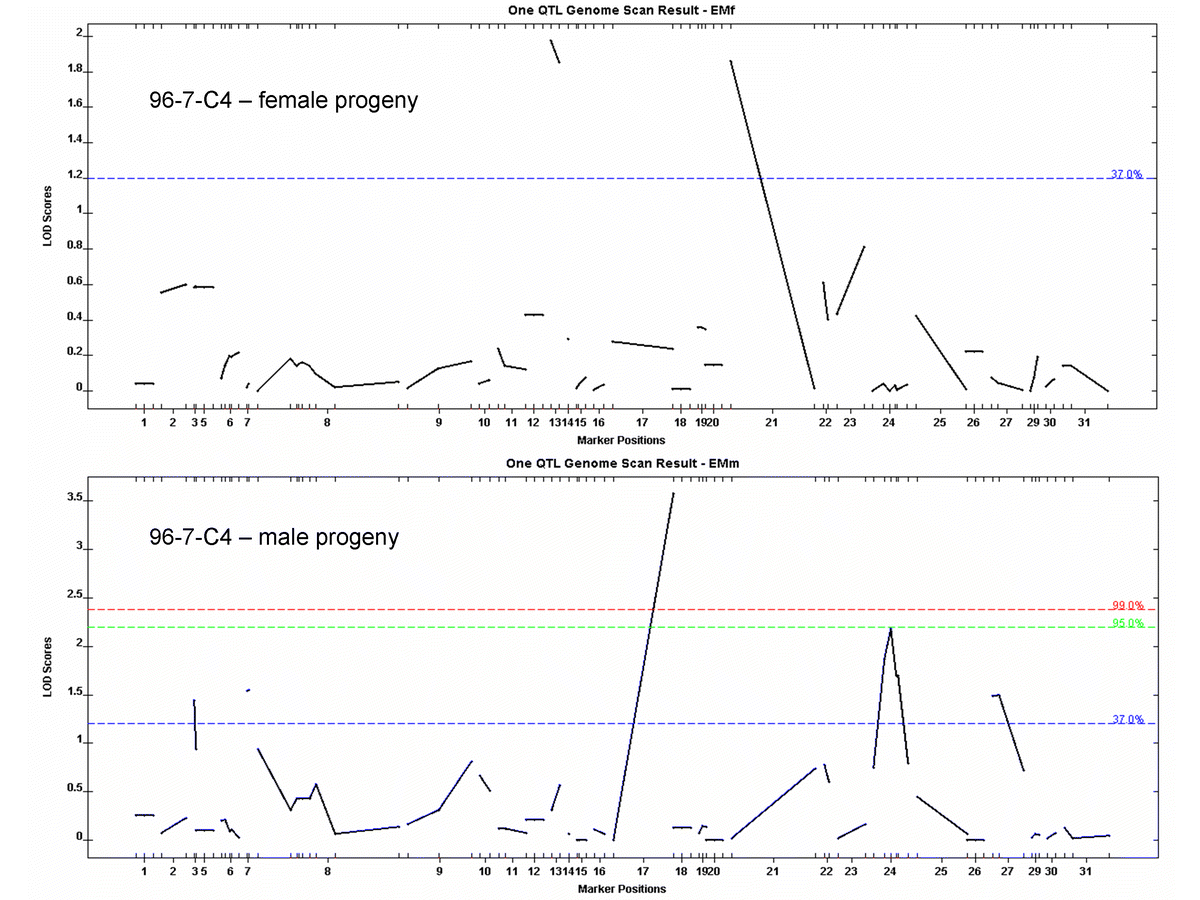

Supplement: Supplementary file 5 [file 10126_2008_9098_Fig1e_ESM.gif]

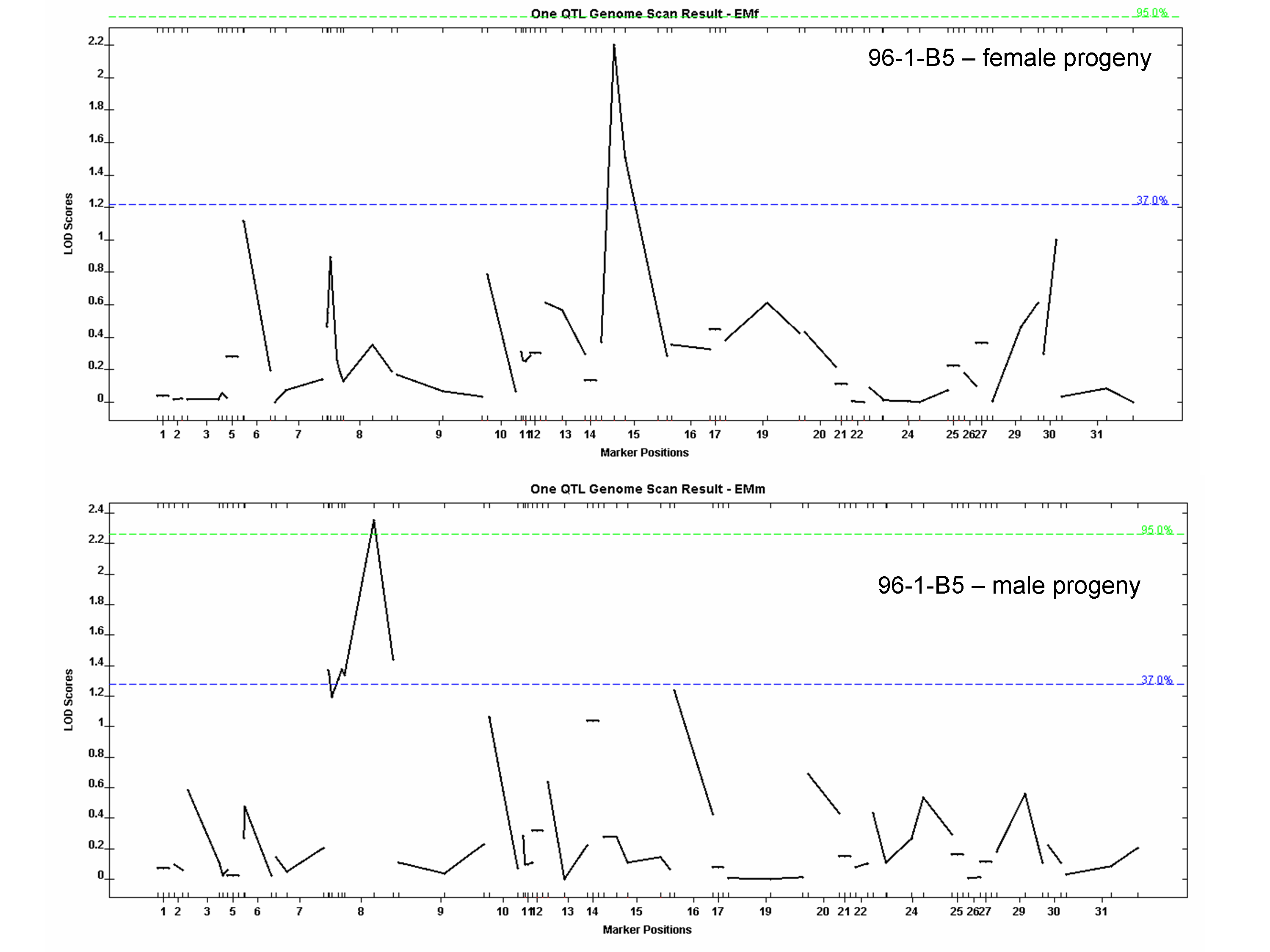

Supplement: Supplementary file 6 [file 10126_2008_9098_Fig1a_ESM.tif]

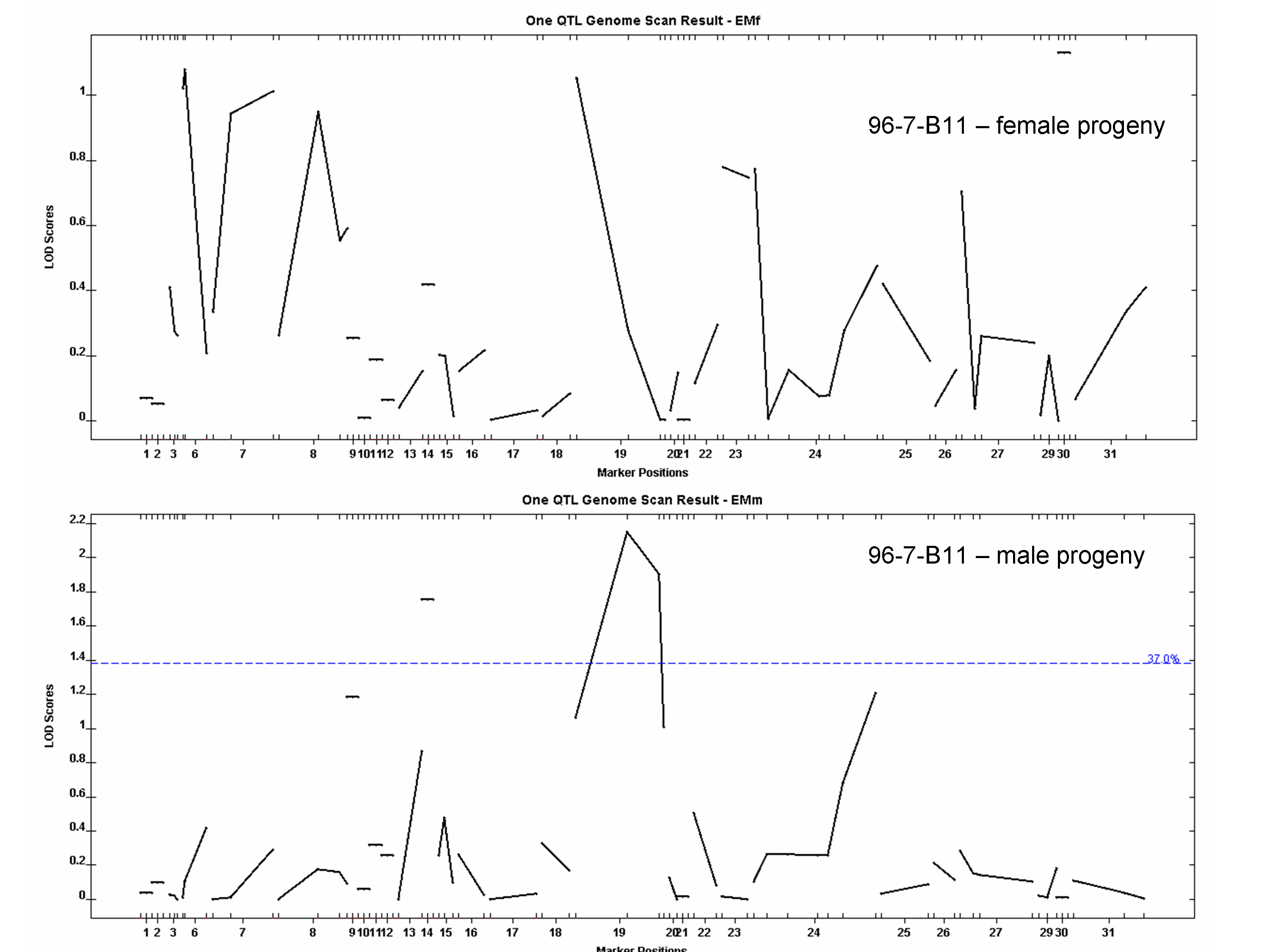

Supplement: Supplementary file 7 [file 10126_2008_9098_Fig1b_ESM.tif]

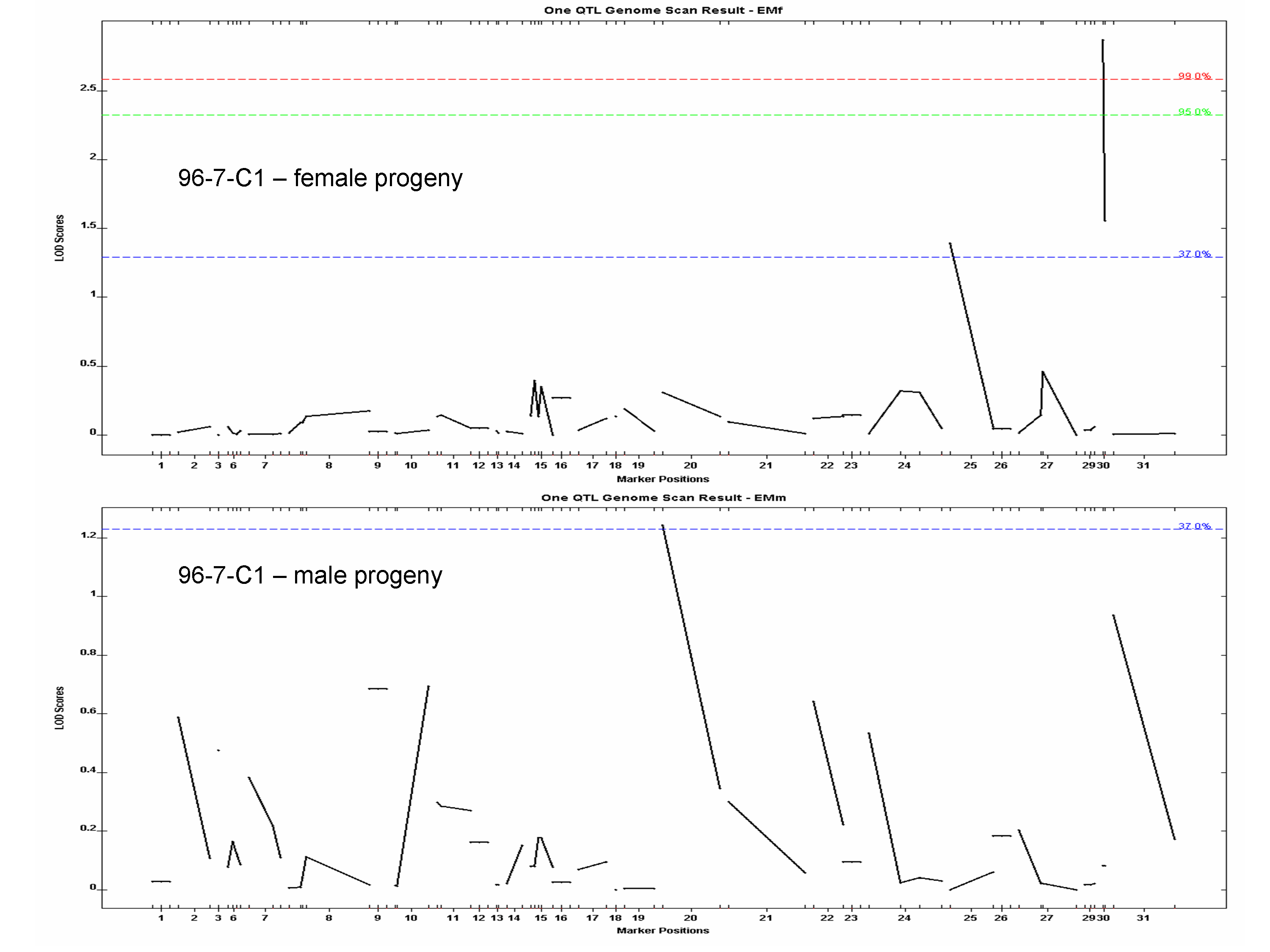

Supplement: Supplementary file 8 [file 10126_2008_9098_Fig1c_ESM.tif]

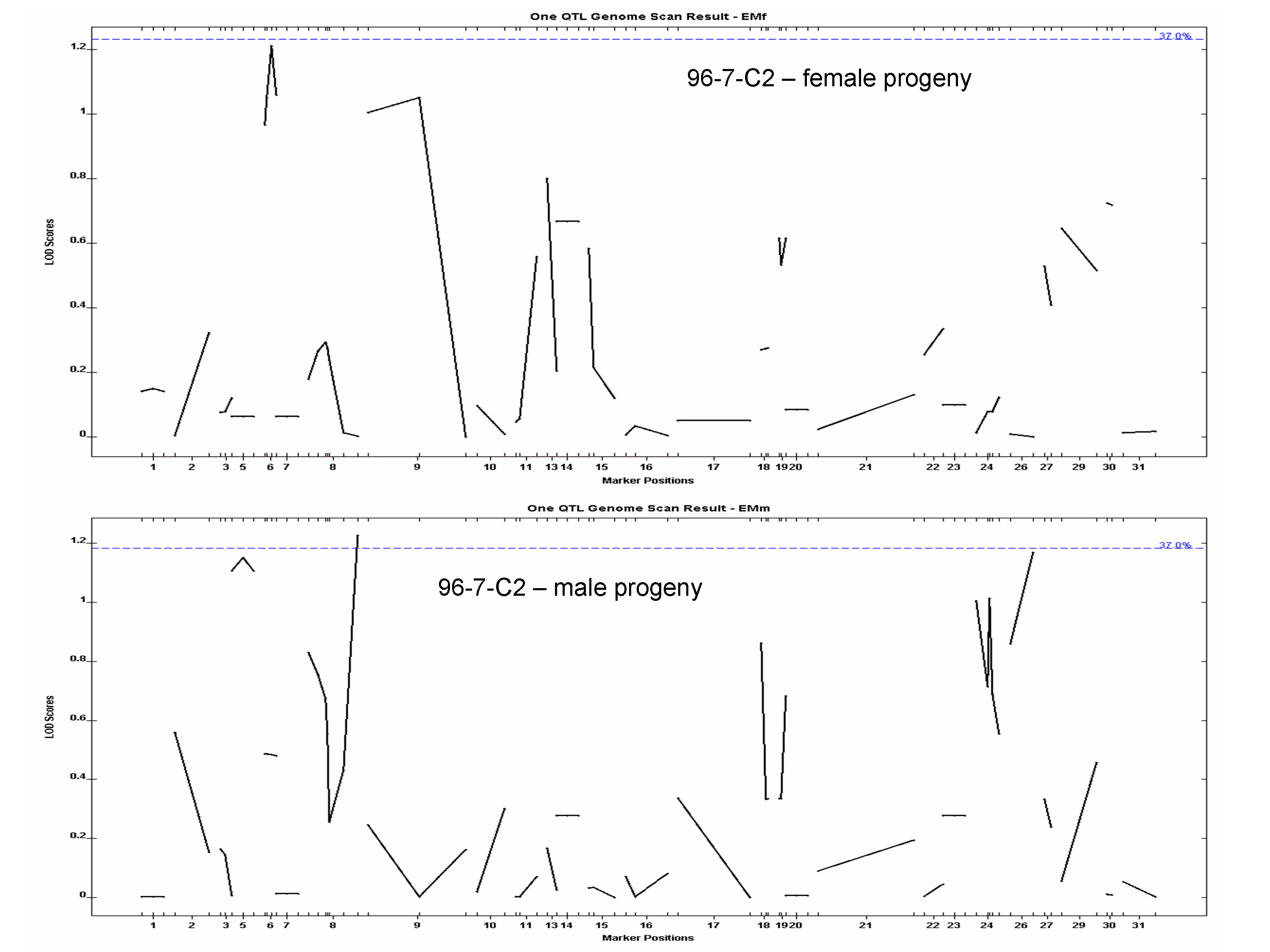

Supplement: Supplementary file 9 [file 10126_2008_9098_Fig1d_ESM.tif]

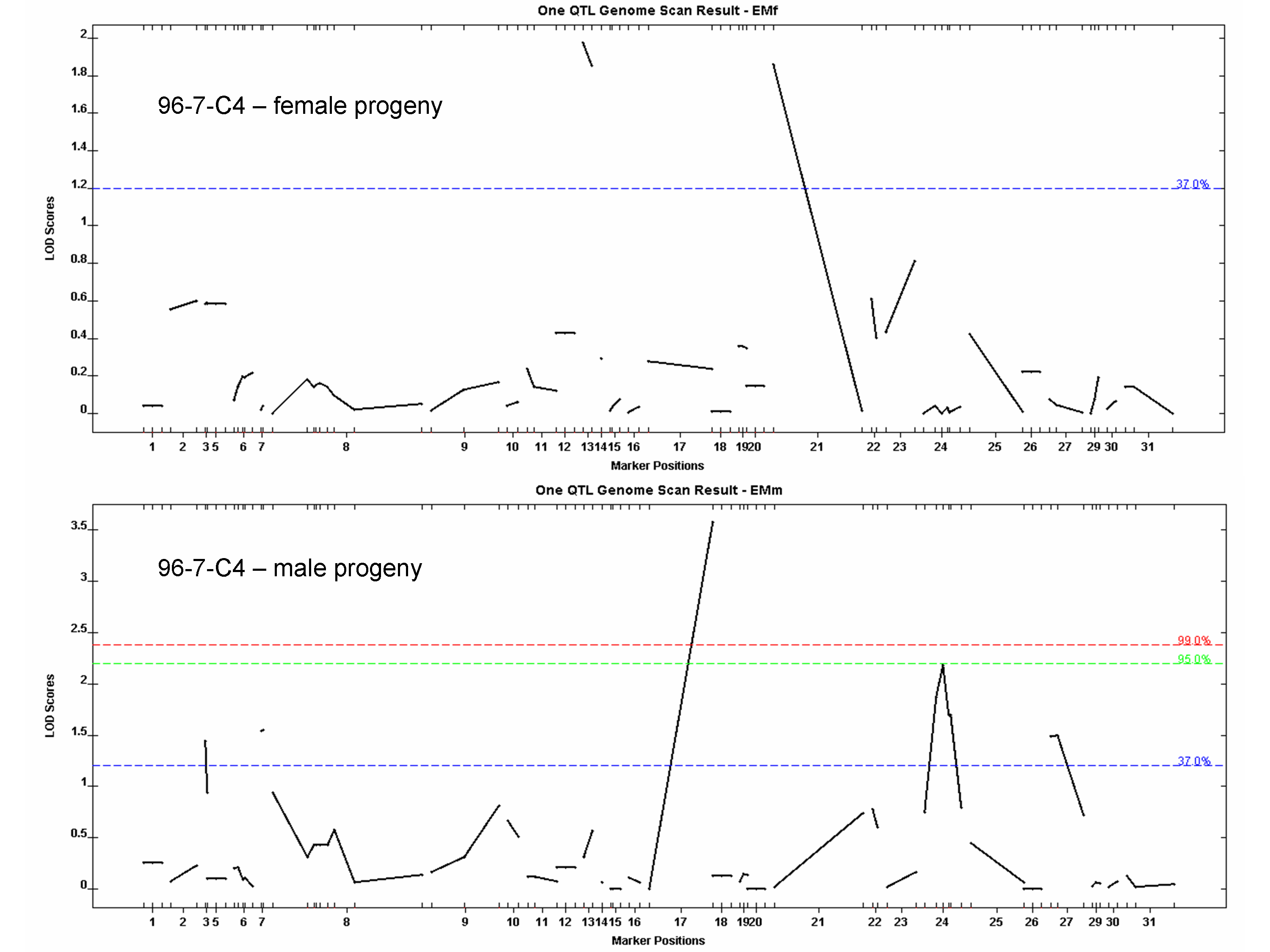

Supplement: Supplementary file 10 [file 10126_2008_9098_Fig1e_ESM.tif]

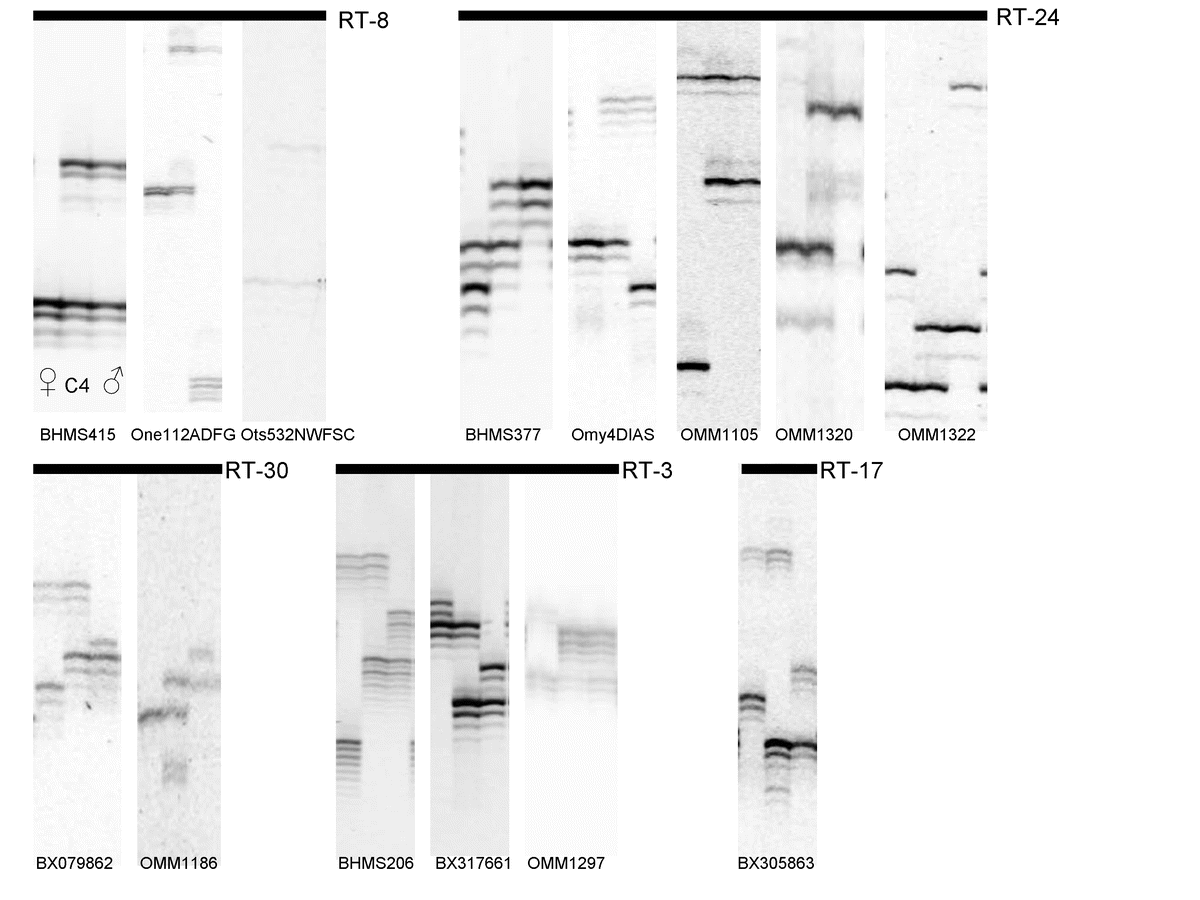

Supplement: Supplementary file 11 — TAMRA-labelled DNA fragments generated for the various EM QTL markers detected in rainbow trout. Associations of marker to linkage groups are indicated by the solid black bar at the top of each set of markers. For each panel, the maternal parent (93-32-3) and paternal parent (94-37-8) of family 96-7-C are shown in the left and rightmost lane, respectively. One of the three full-sib brothers (i.e., 96-7-C4) is shown in the middle lane. Allele sizes (bp) for both of the parents and their three male offspring are given in Table 5. The smallest allele bp sizes are at the bottom of the panel and increase in size in ascending order within each figure. Panels are not represented to a uniform scale (GIF 126 kb) [file 10126_2008_9098_Fig2_ESM.gif]

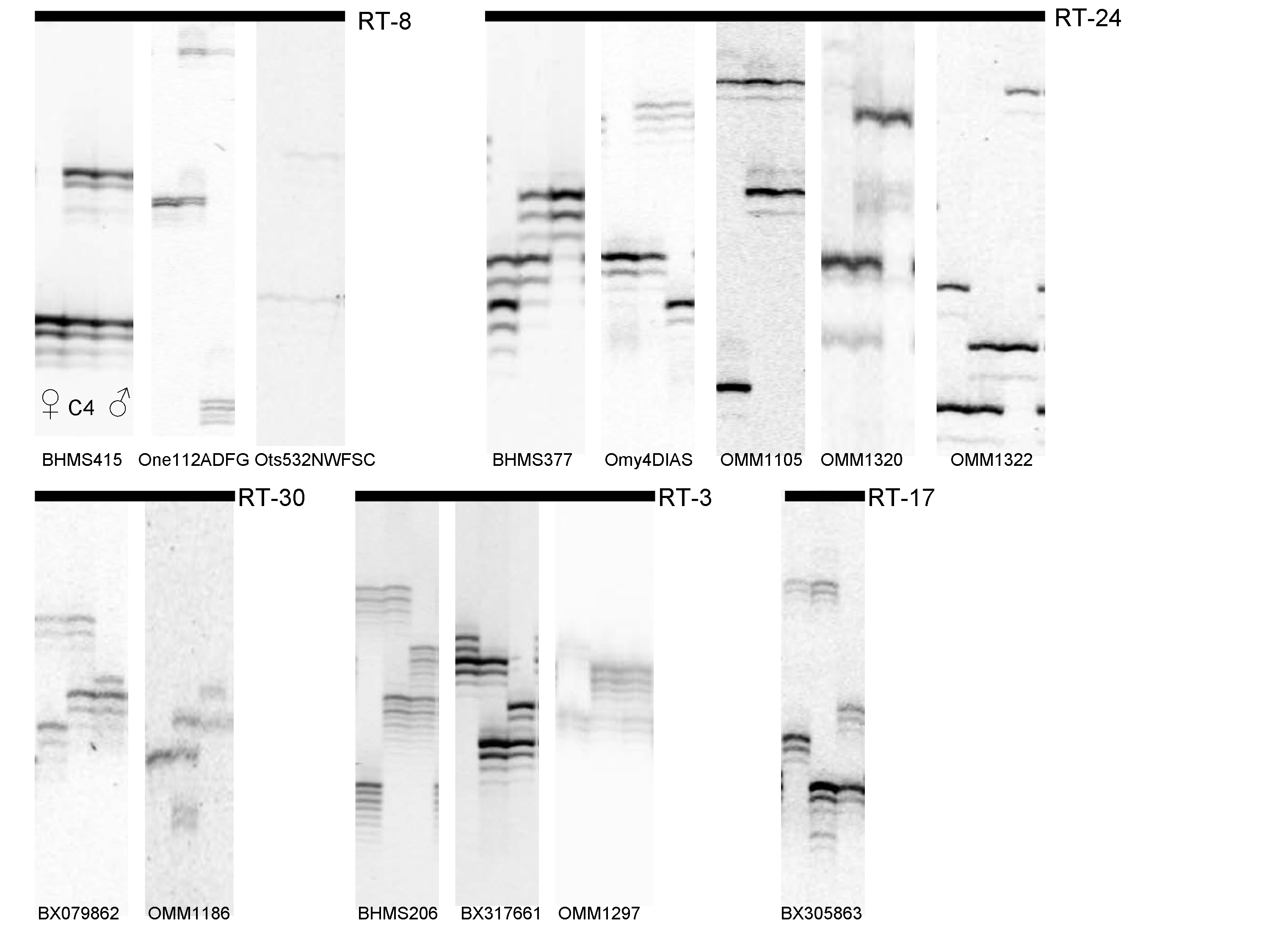

Supplement: Supplementary file 12 [file 10126_2008_9098_Fig2_ESM.tif]

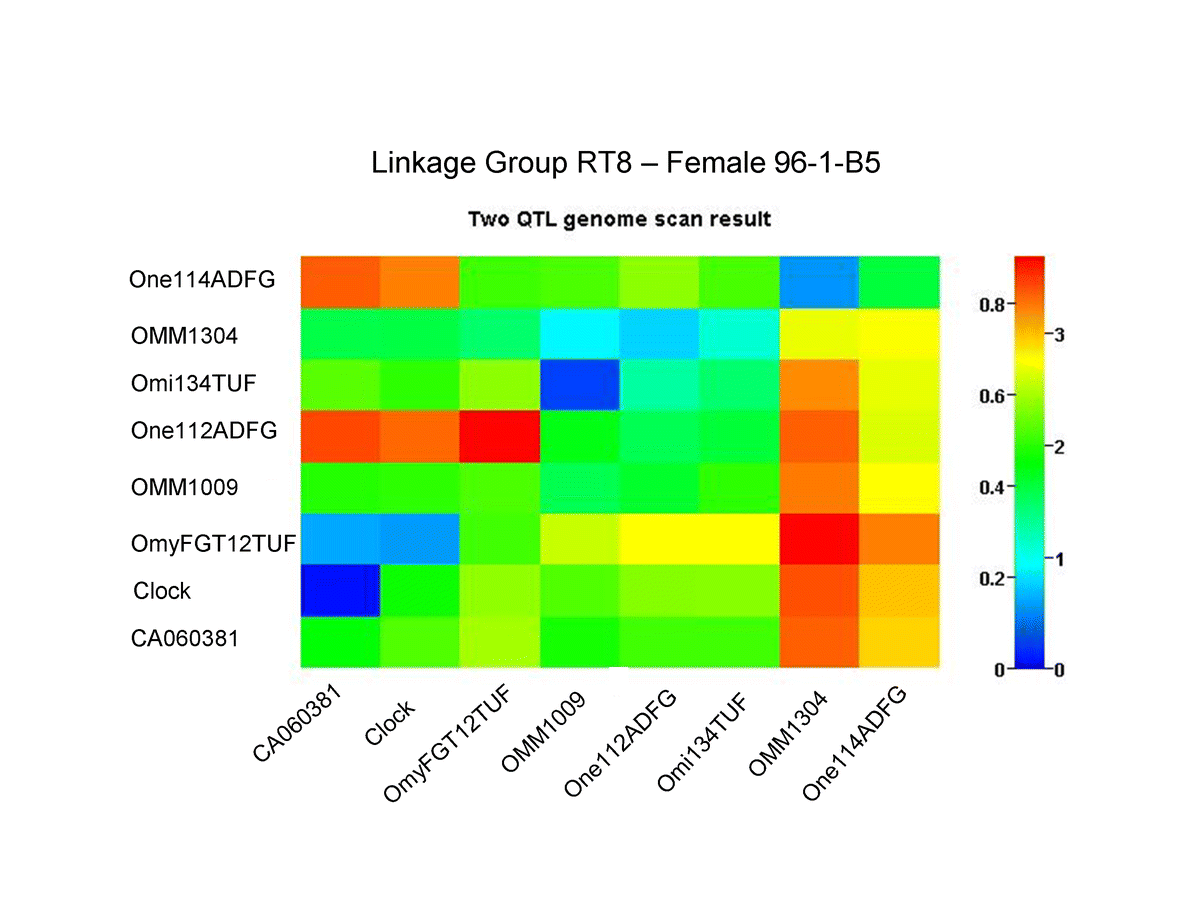

Supplement: Supplementary file 13 — Scantwo R/qtl plots from linkage group RT-8 depicting combined male- and female-specific QTL regions using sex as a co-variate. Epistasis LOD peaks are shown in the upper left of the figure according to the left-hand side of the LOD scale depicted. Additive LOD scores are shown in the lower right with the highest LOD peak (3.678) according to the right-hand side of the LOD scale depicted. The highest LOD score quadrant indicates that two contributing regions (OmyFGT12TUF and OMM1304) are likely present in the genome of this female (GIF 149 kb) [file 10126_2008_9098_Fig3_ESM.gif]

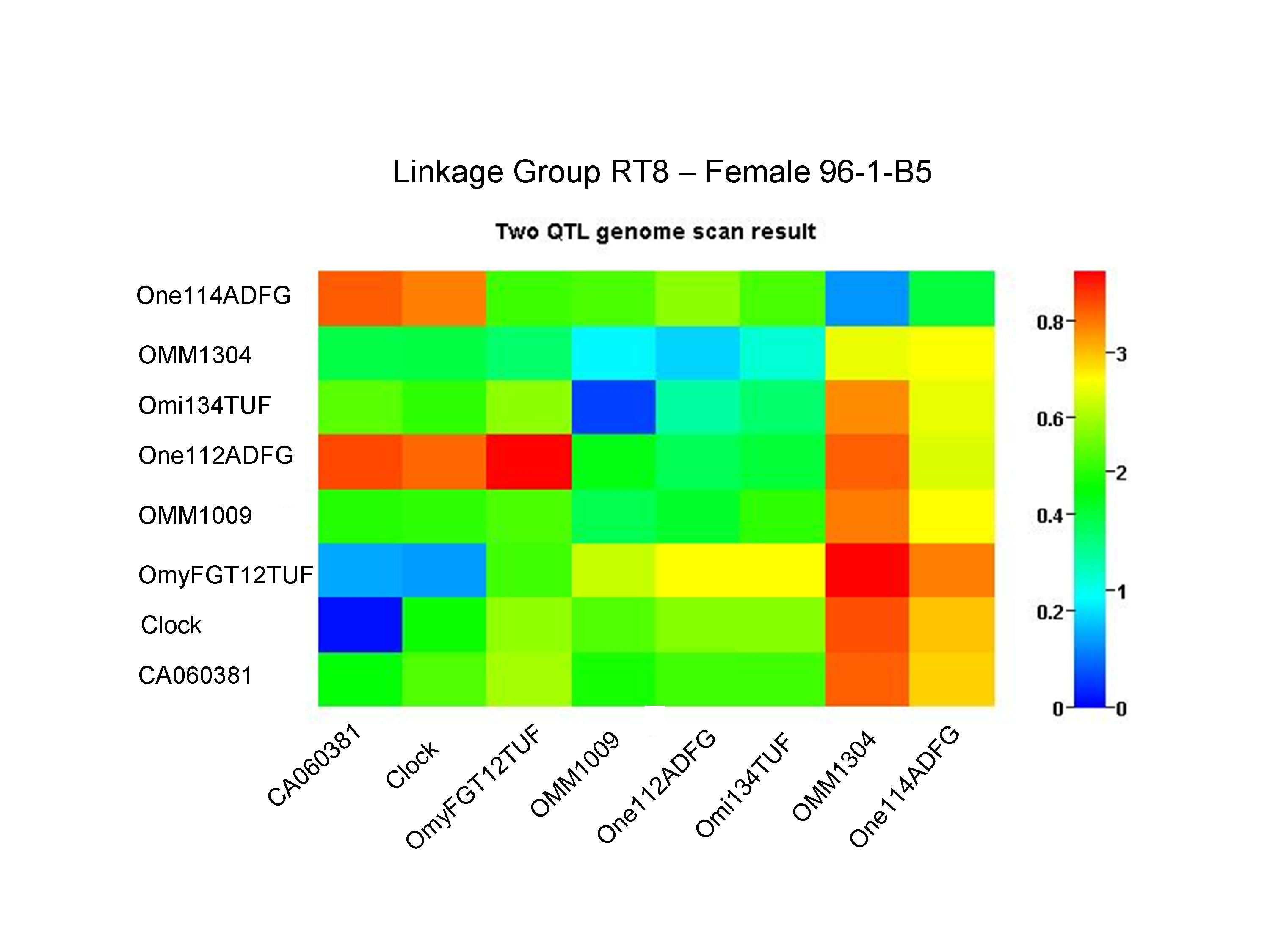

Supplement: Supplementary file 14 [file 10126_2008_9098_Fig3_ESM.tif]
