# Supplementary material for: Determination of Quantitative Trait Loci (QTL) for Early Maturation in Rainbow Trout (Oncorhynchus mykiss)
Source: Mar Biotechnol (NY). 2008 May 20;10(5):579–92. doi: 10.1007/s10126-008-9098-5 (PMC2516301; doi:10.1007/s10126-008-9098-5)
Supplement: Supplementary file 15 [file 10126_2008_9098_MOESM15_ESM.doc]

**Supplementary Table 1.** Microsatellite loci used in a genome scan for early maturation, body weight and condition factor QTL in six diallel families of rainbow trout. The linkage group on which the locus is located, the primer sequence and the annealing temperature (AT ^o^C) are given. (Omy = *Oncorhynchus mykiss,* OMM = *Oncorhynchus mykiss* microsatellite, Ots = *Oncorhynchus tshawytscha*, BHMS = *Salmo salar* clone, SSM = *Salmo salar* microsatellite, DIAS = Danish Institute of Agricultural Science, INRA = Institut National de la Recherche Agronomique, TUF = Tokyo University of Fisheries).

|  | **Microsatellite locus** | **Linkage group** | **Primer sequence (5' to 3')** | **AT** | **Accession** | **Reference** |
| --- | --- | --- | --- | --- | --- | --- |
| 1 | OMM5641 | 1 | AAGACAGGAGATGAAGACACCG | 54 | BX076085 | (Govoroun*, et a*l., 2006) |
|  |  |  | ATATGTCGTGGGAAACATGTAGG |  |  | (Gharbi *et al.* unpublished) |
| 2 | OMM1262/i/ii | 2/9 | AGGTGCAATCTGGCGTTCTG | 60 | AF470028 | (Rexroa*d et a*l., unpublished) |
|  |  |  | AAAGGCGAAGTCGGGTAACAG |  |  |  |
| 3 | OMM6050/i/ii | 2/9 | TTAAGCACTAAGGGGAAGACGG | 56 | CA376300 | (Rexroa*d et a*l., 2003) |
|  |  |  | TGGGGTGGAAGCAAAGAGC |  |  | (Gharbi *et al.* unpublished) |
| 4 | OMM5748/i/ii | 2/29 | ATAGCCAGAGGGAAGCCTGC | 54 | BX087664 | (Govoroun *et al.*, 2006) |
|  |  |  | GCATCTCCAGCAGTCATTTGG |  |  | (Gharbi *et al.* unpublished) |
| 5 | OMM5712/i/ii | 2/29 | TCCGTCTGTTTGTCTCTGTGC | 54 | BX861650 | (Govoroun *et al.*, 2006) |
|  |  |  | GGAATAGGGCTCCTTTTGGG |  |  | (Gharbi *et al.* unpublished) |
| 6 | OMM1162 | 2/29 | GCGAGGGATTGAGGGATTGA | 58 | AY039644 | (Rexroa*d et a*l., 2002a) |
|  |  |  | TCTGCACCCTCCTTTTACCCA |  |  |  |
| 7 | OMM5639 | 3 | GACTACATACAATGGGTCGAAGC | 56 | BX317661 | (Govoroun, *et al.*, 2006) |
|  |  |  | GCTGGAATTCAGAGTGCTAGAGC |  |  | (Gharbi *et al.* unpublished) |
| 8 | BHMS206 | 3 | CCAAATAACTGACAAGTGAG | 54 | AF256680 | (Høyheim, unpublished) |
|  |  |  | CAGAGGTTGATAATGGGG |  |  |  |
| 9 | OMM1230 | 3 | CGTGACACTGATTGGCA | 58 | AF470010 | (Rexroad *et al.*, unpublished) |
|  |  |  | CACACGCTCGCTCTATTT |  |  |  |
| 10 | OMM1297 | 3 | GGAGGGGAAATGAGGGAAGAGTAT | 60 | AF470055 | (Rexroad *et al.*, unpublished) |
|  |  |  | CAGAACAGGGGATGAGAAGAGAGTAAG |  |  |  |
| 11 | OMM5672/i/ii | 5/31 | GATTTTCTGACCAGCACCTCC | 56 | BX318599 | (Govoroun, *et al.*, 2006) |
|  |  |  | CCAACTGGACCCAGAACAGC |  |  | (Gharbi *et al.* unpublished) |
| 12 | OMM1082 | 6 | CAAGAGCACTAACGACCATGT | 56 | AF352753 | (Rexroad*, et a*l., 2002b) |
|  |  |  | CGCAAGCAAGCTAACACA |  |  |  |
| 13 | OMM1355 | 6 | ATCTGGTCTGTTGGAAGTCAG | 56 | BV005147 | (Palt*i et a*l., 2002a) |
|  |  |  | GGGATTTAGGAGAGACGTT |  |  |  |
|  |  |  |  |  |  |  |
| Appendix 1 continued | |  |  |  |  |  |
|  | **Microsatellite Locus** | **Linkage group** | **Primer sequence (5' to 3')** | **AT** | **Accession** | **Reference** |
| 14 | OMM1205 | 6 | AAACGGTGCCCTCCTTCCTCTATA | 56 | AF469990 | (Rexroad *et al.*, unpublished) |
|  |  |  | CCCAAGCCAATAAAGCCCTTACAT |  |  |  |
| 15 | OMM1302 | 6 | AGCCAGCCAATTAATACCCTG | 56 | G73542 | (Palt*i et a*l., 2002b) |
|  |  |  | TTCTGTGTGGCCTAAACCTT |  |  |  |
| 16 | OMM1359 | 6 | TTGAAGAAGGGCGAGAG | 56 | BV005151 | (Palti *et al.*, 2002a) |
|  |  |  | AGACATGGGGCAGTCTAGAAG |  |  |  |
| 17 | OMM1034 | 7 | ACCCCCGCCCAGTCGTCTCTCT | 58 | AF346684 | (Rexroad*, et a*l., 2002c) |
|  |  |  | TTGGGGGTGTCTTGCTAATTGCCT |  |  |  |
| 18 | OMM1087 | 7 | GACGCAGAAGTGTTTAGCTCT | 56 | AF352756 | (Rexroad, *et al.*, 2002b) |
|  |  |  | TTACTGTCTTCTCGCAGCA |  |  |  |
| 19 | OMM1305 | 7 | AATTGGCACCCTCTCGTCTG | 56 | G73545 | (Palti *et al.*, 2002b) |
|  |  |  | AAACAGGGAGTGTGGCACAAA |  |  |  |
| 20 | OMM1009 | 8 | ACTGGAATCCAATAACAACCC | 58 | AF346671 | (Rexroad, *et al.*, 2002c) |
|  |  |  | CGGAGGTTTGATGAGTCATT |  |  |  |
| 21 | Ots532NWFSC | 8 | TCATTATCTGATTTACTACACAG | 50 | AY042722 | (Naish and Park, 2002) |
|  |  |  | TTATGCCTGGTCTGGAAC |  |  |  |
| 22 | OMM1304 | 8 | ATAGATGTAAAGACAGAGCGAGAC | 54 | G73544 | (Palti *et al.*, 2002b) |
|  |  |  | GCAGAGAGGAAATCGGTGA |  |  |  |
| 23 | OmyFGT12TUF | 8 | CAGTGTTGGAACACGTCCTG | 56 |  | (Sakamoto, unpublished) |
|  |  |  | TTGATTCTTGTGATGAAATCGC |  |  |  |
| 24 | BHMS415 | 8 | CAGATTTTGGGCAAGATTC | 54 | AF256747 | (Høyheim, unpublished) |
|  |  |  | ATGAGAGGGGACAGAAGC |  |  |  |
| 25 | SSM1025 | 8 | AGTGGTGAAGTGGGATGGGG | 56 | CA060391 | (Brierley and Williams, unpublished) |
|  |  |  | CCCGATGCTTTCTTCATGG |  |  | (Gharbi *et al.* unpublished) |
| 26 | Clock | 8 | ATAGGTTACCTGCCGTTTGA | 54 | AF266745 | (Mazurai*s et a*l., unpublished) |
|  |  |  | ATTTCCCCTTCCCATACTGCA |  |  |  |
| 27 | Omi134TUF | 8 | ATACCACATTAATGCATTCCCC | 54 | AB105851 | (Har*a et a*l., unpublished) |
|  |  |  | GAGCAGGACGGAGAGAGATG |  |  |  |
| 28 | One112ADFG | 8 | GTGACCCAGACTCAGAGGAC | 54 | AF274528 | (Olsen *et al.*, 2000) |
|  |  |  | CACAACCCATCACATGAAAC |  |  |  |
| 29 | One114ADFG | 8 | TCATTAATCTAGGCTTGTCAGC | 54 | AF274530 | (Olsen *et al.*, 2000) |
|  |  |  | TGCAGGTAAGACAAGGTATCC |  |  |  |
|  |  |  |  |  |  |  |
|  |  |  |  |  |  |  |
| Appendix 1 continued | |  |  |  |  |  |
|  | **Microsatellite Locus** | **Linkage group** | **Primer sequence (5' to 3')** | **AT** | **Accession** | **Reference** |
| 30 | SMM1016 | 9 | GACATACTGACACAGGGTTCACG | 58 | CB497405 | (Ris*e et a*l., 2004) |
|  |  |  | GACACAGGATTTGATTTGGCTC |  |  | (Gharbi *et al.* unpublished) |
| 31 | OmyRGT30TUF | 9 | GATCCGTGTGAGTGATGTGG | 54 | AB087600 | (Sakamoto & Ozaki, unpublished) |
|  |  |  | GAATGAGTTGCAAGCAGGC |  |  |  |
| 32 | OMM5780 | 10 | TCCGCAACAAGTACGCTGG | 54 | CR363293 | (Govoroun *et al.*, 2006) |
|  |  |  | TTCTCTTCTGGCAACTTCAGACC |  |  | (Gharbi *et al.* unpublished) |
| 33 | OMM1179 | 10 | TGGAGGGGTTTTSSTGGCAC | 58 | AF469966 | (Rexroad *et al.*, unpublished) |
|  |  |  | TGACAGAGGTGGGGAGATTGA |  |  |  |
| 34 | Omy1225UW | 10 | CTTCCCGCACTCACACTGTTTTTG | 56 |  | (Spie*s et a*l., 2005) |
|  |  |  | TGATTTAGAGGCCAAGAAGCACTGG |  |  |  |
| 35 | OMM5638 | 11 | CATGGAGTGTAAGGCAGGGC | 56 | BX313739 | (Govoroun *et al.*, 2006) |
|  |  |  | CGACAGATCAGCATCGCTGC |  |  | (Gharbi *et al.* unpublished) |
| 36 | OMM1315 | 11 | TACAGGGCTTGGCTCTATCTC | 56 | G73554 | (Palti *et al.*, 2002b) |
|  |  |  | GCCAAATACTTTCGCAAGG |  |  |  |
| 37 | Omy1011UW | 11 | AACTTGCTATGTGAATGTGC | 56 | AY518334 | (Spie*s et a*l., 2005) |
|  |  |  | GACAAAAGTGACTGGTTGGT |  |  |  |
| 38 | Ots515NWFSC | 11 | ACAGTGATGGAGCTTGATTC | 54 | AY042705 | (Naish and Park, 2002) |
|  |  |  | ACGATTTCTATTTGTCTCCG |  |  |  |
| 39 | OMM5629 | 12/16 | GTGACATTACAAATCAAGTGCTCC | 58 | CA349039 | (Rexroad *et al.*, 2003) |
|  |  |  | CTAAATGGTGAGTACGATGCCC |  |  | (Gharbi *et al.* unpublished) |
| 40 | OmyRGT14TUF | 13 | CCTGGCTCTGTTACCTGTCTG | 58 | AB087593 | (Sakamoto & Ozaki, unpublished) |
|  |  |  | ATCAATAAACCGCAAATGGG |  |  |  |
| 41 | OMM1216 | 13 | TCATGCTAACCACTAACCAACC | 56 | AF469998 | (Rexroad *et al.*, unpublished) |
|  |  |  | CGGAATGTGGACAAGATCAG |  |  |  |
| 42 | OMM1321 | 13 | GACCATGCTATCCTATTAAGCTGA | 58 | G73559 | (Palti *et al.*, 2002b) |
|  |  |  | GGGGAGATGGATAAGGAGATA |  |  |  |
| 43 | OMM5705 | 14 | AGAGAACCAGTGACCCAGCC | 54 | BX080247 | (Govoroun *et al.*, 2006) |
|  |  |  | GCAACCTACCCCTTCATCCC |  |  | (Gharbi *et al.* unpublished) |
|  |  |  |  |  |  |  |
|  |  |  |  |  |  |  |
|  |  |  |  |  |  |  |
| Appendix 1 continued | |  |  |  |  |  |
|  | **Microsatellite Locus** | **Linkage group** | **Primer sequence (5' to 3')** | **AT** | **Accession** | **Reference** |
| 44 | OMM1657 | 14/20 | CAAAAGGGGATTGTTGTACG | 56 | BV212158 | (Coulibaly *et al*. 2005) |
|  |  |  | GCTCCAGGACTTTGTTCAGA |  |  |  |
| 45 | OMM1134 | 14/20 | GAAGTTCATCTCCAGGTCAAACTG | 56 |  | (Rexroad et al., unpublished) |
|  |  |  | TGCGTAGGTTGATGAATCCTC |  |  |  |
| 46 | OMM1036 | 15 | TGTAGCAGGTGAGAAATACCCA | 56 | AF346686 | (Rexroad *et al.*, 2002c) |
|  |  |  | CACCATCTCCATCCTAGCC |  |  |  |
| 47 | OMM1051 | 15 | CCTACAGTAGGGATTAACAGC | 56 | AF346695 | (Rexroad *et al.*, 2002c) |
|  |  |  | CATGCCCACACATTACTAC |  |  |  |
| 48 | OMM1166 | 15 | TTCGCAGGACCCATCTCTGTAGTCGTC | 56 | AY039646 | (Rexroad *et al.*, 2002a) |
|  |  |  | GGCTCAGGCATGGCATTCACTAAGGA |  |  |  |
| 49 | OMM1175 | 15 | CTCTCACCAGGCAATTAGTCT | 56 | AF469963 | (Rexroad *et al.*, unpublished) |
|  |  |  | CCTCTCCCTCCTACTGTCC |  |  |  |
| 50 | OMM1260 | 15 | CGCTGTTGACATGACCTTAC | 56 | AF470027 | (Rexroad *et al.*, unpublished) |
|  |  |  | GGAGGTGGTAGACGATACAGA |  |  |  |
| 51 | OmyRGT23TUF | 15 | TCCAGAGGGGGCTGTGTT | 54 |  | (Kho*o et a*l., 2000) |
|  |  |  | CTTTCTTTGCTGCACTTGACC |  |  |  |
| 52 | OMM5607 | 16 | ACCACCTCAGAGTGTCTTTCTCC | 58 | CA345149 | (Rexroad *et al.*, 2003) |
|  |  |  | GACTTGACATACAAACGACGTGG |  |  | (Gharbi *et al.* unpublished) |
| 53 | OMM5689 | 16 | GACCTCTCCATCAAGAGCCC | 54 | BX867838 | (Govoroun *et al.*, 2006) |
|  |  |  | GGTGGATGTGGTATGTGTTGC |  |  | (Gharbi *et al.* unpublished) |
| 54 | OMM1090 | 17 | TGCGGTAGGAAGGCTTTAGTG | 56 | AF352759 | (Rexroad *et al.*, 2002b) |
|  |  |  | AAATGGAGCAGCGCTGGTAT |  |  |  |
| 55 | OMM5667 | 17 | TACTGTACAGGATGGGTCTCTGC | 58 | BX305863 | (Govoroun *et al.*, 2006) |
|  |  |  | GGAGTACTACGTGTGAGGATTGG |  |  | (Gharbi *et al.* unpublished) |
| 56 | Omi87TUF | 18 | CCAACTCCCGTATCCTCAGA | 58 | AB213231 | (Hara, *et al.*, unpublished) |
|  |  |  | TGCCTTTCAGAAGGTGGC |  |  |  |
| 57 | OMM1311 | 18 | CCCAGTGCCACTCAAGTTCA | 58 | G73551 | (Palti *et al.*, 2002b) |
|  |  |  | GTGCAGAGAAAGCCACGATTA |  |  |  |
| 58 | OmyRGT12TUF | 18 | TGAAGACGTTGTGGCTCCTA | 58 | AB087591 | (Sakamoto *et al.*, 2000) |
|  |  |  | CAAAGCACCTGGCCTGTAAT |  |  |  |
|  |  |  |  |  |  |  |
| Appendix 1 continued | |  |  |  |  |  |
|  | **Microsatellite Locus** | **Linkage group** | **Primer sequence (5' to 3')** | **AT** | **Accession** | **Reference** |
| 59 | OmyCosBTUF | 18 | CCAGAACTATGACTACCCAGAG | 54 |  | (Sakamoto *et al*., unpublished) |
|  |  |  | GTTACAGAGCTGCCACAGTATT |  |  |  |
| 60 | OMM5707 | 19 | AAAAGATCAGCCGCAAGCC | 54 | BX298853 | (Govoroun *et al.*, 2006) |
|  |  |  | GGGTGCTGTAATTAAATCGGC |  |  | (Gharbi *et al.* unpublished) |
| 61 | SSM1004 | 19 | GATCCACACAAGTTTGGACTGG | 54 | CA058586 | (Rise *et al.*, 2004) |
|  |  |  | CTGCAGGGGTGAATTGTCC |  |  | (Gharbi *et al.* unpublished) |
| 62 | OMM1025 | 19 | GCGCATTGTAGTCTCGTC | 56 | AF346682 | (Rexroad *et al.*, 2002c) |
|  |  |  | AGTCCGCTATGTTGTTATGTA |  |  |  |
| 63 | Omy103INRA | 19 | TGGAAGCAGTCAAGGTACGTA | 58 |  | (Danzmann *et al.* 2005) |
|  |  |  | TGTATGCACTAATAGGCCTTT |  |  |  |
| 64 | OMM5721 | 20 | GGTTCTGGCCCGTTATCTGG | 54 | BX882100 | (Govoroun *et al.*, 2006) |
|  |  |  | TTGCCTGGAGAAAGTGCCG |  |  | (Gharbi *et al.* unpublished) |
| 65 | OMM1257 | 20 | AAATTGTGTGTCTGCGTGCTTG | 56 | AF470024 | (Rexroad *et al.*, unpublished) |
|  |  |  | TAGGTGAGGAACACAGCCTACCTG |  |  |  |
| 66 | OMM5750 | 21 | ACACCTCTACGACATGGAGGC | 56 | BX302949 | (Govoroun *et al.*, 2006) |
|  |  |  | GTGTGTCTGCTGGTTAATTCCC |  |  | (Gharbi *et al.* unpublished) |
| 67 | OMM1059 | 21 | CGCCAGATGATTAAACGA | 58 | AF352742 | (Rexroad *et al.*, 2002b) |
|  |  |  | GGGCTATTCACACGTTCA |  |  |  |
| 68 | OMM5640 | 22 | CGTCATCCATGTATGTTTCATGC | 54 | BX319197 | (Govoroun *et al.*, 2006) |
|  |  |  | GACAACTCACACATCCACATGG |  |  | (Gharbi *et al.* unpublished) |
| 69 | OMM5677 | 22 | AGGCTGCGTGTAAGAGCTCC | 56 | BX860095 | (Govoroun, *et al.*, 2006) |
|  |  |  | CAAAAGTCATGAGGACCGCC |  |  | (Gharbi *et al.* unpublished) |
| 70 | OMM1055 | 23 | GGCCCAAAGTCTTTAGTGTTG | 58 | AF352740 | (Rexroad *et al.*, 2002b) |
|  |  |  | GCCCAGCTTACTCTCATTACC |  |  |  |
| 71 | OMM1097 | 23 | CTAGCCATCCGAACACTG | 56 | AF352763 | (Rexroad *et al.*, 2002b) |
|  |  |  | AGAATAGGGTGCCTGTATCTC |  |  |  |
| 72 | OMM1105 | 24 | GCACACTGTCTGGGTAAGAGA | 58 | AF352768 | (Rexroad *et al.*, 2002b) |
|  |  |  | GCAGAGCCACACTAAACCA |  |  |  |
|  |  |  |  |  |  |  |
|  |  |  |  |  |  |  |
|  |  |  |  |  |  |  |
| Appendix 1 continued | |  |  |  |  |  |
|  | **Microsatellite Locus** | **Linkage group** | **Primer sequence (5' to 3')** | **AT** | **Accession** | **Reference** |
| 73 | BHMS377 | 24 | TGGCTACAACAGGGATAC | 58 | AF256707 | (Høyheim, unpublished) |
|  |  |  | AGTCTCTTACATGGAGGC |  |  |  |
| 74 | Omy27INRA | 24 | CCAATCACCATCTGCTGGG | 58 |  | (Gharb*i et a*l. 2006) |
|  |  |  | GCCCATCGTTTAGCCAGG |  |  |  |
| 75 | Omy4DIAS | 24 | GGGTTTGTGTGCACTGGAG | 54 | AF090389 | (Holm and Brusgaard, 1999) |
|  |  |  | AGCCAGGCTGGTTATGATCT |  |  |  |
| 76 | OmyRGT36TUF | 24 | ATCATCAAGAGTCCATCAGAGC | 58 | AB087605 | (Sakamoto *et al.*, 2000) |
|  |  |  | GGTCTGGTGCCATTCTGG |  |  |  |
| 77 | OMM1320 | 24 | GAAAGTGTCTGTCTGTCCGC | 58 | G73558 | (Palti *et al.*, 2002b) |
|  |  |  | GGTGAATACTTTCGCAAGCCA |  |  |  |
| 78 | OMM1322 | 24 | GCGCTCCTTTCATCTCTGATACAG | 62 | G73560 | (Palti *et al.*, 2002b) |
|  |  |  | GGTGAATACTTTCGCAAGCC |  |  |  |
| 79 | OMM1193 | 25 | CACAATGGCAGCTTAGGGAC | 56 | AF469978 | (Rexroad *et al.*, unpublished) |
|  |  |  | GGCAGCTTGACAAATAACACG |  |  |  |
| 80 | Omy1552UW | 25 | GTAGGACGATGCCACAGTAGTTACAATCAC | 56 |  | (Bentzen, unpublished) |
|  |  |  | CCCTCAGGACATTAGGAATGCCAG |  |  |  |
| 81 | OMM1015 | 26 | GACAAATTCACCCTCTTCTG | 58 | AF346675 | (Rexroad *et al.*, 2002c) |
|  |  |  | CATGAGAACTGTTCCCA |  |  |  |
| 82 | OMM5788 | 26 | CACTGACGAAGTCCAAGTGTACC | 54 | CR375500 | (Govoroun *et al.*, 2006) |
|  |  |  | GCGGAGTGCAGCTTTTAGTCC |  |  | (Gharbi *et al.* unpublished) |
| 83 | OMM5652 | 27 | GGTAATGCAATTTCCCAAGCC | 56 | BX073867 | (Govoroun *et al.*, 2006) |
|  |  |  | ATGACATAGATGGAAAGAAAACGG |  |  | (Gharbi *et al.* unpublished) |
| 84 | OMM5681 | 27 | GTGACCCCAAACTGAACGG | 54 | BX863380 | (Govoroun *et al.*, 2006) |
|  |  |  | CGATCTCATTATCCGTCAGGG |  |  | (Gharbi *et al.* unpublished) |
| 85 | OMM5611 | 27 | AAGTCAGAGCCCATCTCCCC | 58 | CA374878 | (Rexroad *et al.*, 2006) |
|  |  |  | TGGACGAGGAGTGGAAGTCC |  |  | (Gharbi *et al.* unpublished) |
| 86 | OMM1228 | 27 | CCCTTCCTGTGTGTCGTTGTT | 58 | AF470009 | (Rexroad *et al.*, unpublished) |
|  |  |  | CAGGAGTCACTTGGCAGTAGGAG |  |  |  |
| 87 | OtsG43UCD | 29 | AACTCCCGTTGACAATTTACTGTTG | 58 | AF393186 | (Williams*on et a*l., 2002) |
|  |  |  | TTTTGGCAAAGTTGGCTACTCTG |  |  |  |
|  |  |  |  |  |  |  |
| Appendix 1 continued | |  |  |  |  |  |
|  | **Microsatellite Locus** | **Linkage group** | **Primer sequence (5' to 3')** | **AT** | **Accession** | **Reference** |
| 88 | OMM5613 | 30 | TGTGAGAAGAACACGAGAGTTGG | 56 | BX079862 | (Govoroun *et al.*, 2006) |
|  |  |  | GAATGAGGTGTTAGAACGACTGC |  |  | (Gharbi *et al.* unpublished) |
| 89 | OMM1186 | 30 | CTGCAGAAGGGAAAGAAAGATGGA | 60 | AF469971 | (Rexroad *et al.*, unpublished) |
|  |  |  | TGGGGTGAACGTTGATCTGG |  |  |  |
| 90 | OMM1337 | 31 | TACCTGCGTTAACTATCAATTCAG | 58 | G73570 | (Palti *et al.*, 2002b) |
|  |  |  | CTTCTCTCATACCGCTAAA |  |  |  |
| 91 | OmyRGT1TUF | 31 | AGTTTTGATTGAACGGGGC | 58 | AB087586 | (Sakamoto *et al.*, 2000) |
|  |  |  | CAGGGGACGCCACCTATAC |  |  |  |

**References**

Coulibaly, I., Gharbi, K., Danzmann, R.G., Yao, J., and Rexroad, C.E. III, 2005. Characterization and comparison of microsatellites derived from repeat-enriched libraries and expressed sequence tags. Animal Genetics 36, 309-315.

Danzmann, R., Cairney, M., Davidson, W., Ferguson, M., Gharbi, K., Guyomard, R., Holm, L., Leder, E., Okamoto, N., Ozaki, A., Rexroad, C.I., Sakamoto, T., Taggart, J., and Woram, R., 2005. A comparative analysis of the rainbow trout genome with 2 other species of fish (Arctic charr and Atlantic salmon) within the tetraploid derivative Salmonidae family (subfamily: Salmoninae). Genome 48, 1037-1051.

Gharbi K, Gautier A, Danzmann RG, Gharbi S, Sakamoto T, Hoyheim B, Taggart JB, Cairney M, Powell R, Krieg F, Okamoto N, Ferguson MM, Holm LE, and Guyomard R, 2006. A linkage map for brown trout (*Salmo trutta*): chromosome homeologies and comparative genome organization with other salmonid fish. Genetics 172, 2405-2419.

Govoroun, M., LeGac, F., and Guiguen, Y., 2006. Generation of a large scale repertoire of expressed sequence tags (ESTs) from normalized rainbow trout cDNA libraries. BMC Genomics 7, 126.

Holm, L.E., Brusgaard, K., 1999. Two polymorphic dinucleotide repeats in rainbow trout (*Oncorhynchus mykiss*). Animal Genetics 30 (2), 162-163.

Khoo, S., Ozaki, A., Sakamoto, T., Okamoto, N., 2000. Rapid communication: Two highly polymorphic dinucleotide microsatellites in rainbow trout (*Oncorhynchus mykiss*): OmyRGT18TUF and OmyRGT23TUF. Journal of Animal Science 78, 490-491.

Naish, K., Park, L.K., 2002. Linkage relationships for 35 new microsatellite loci in Chinook salmon *Oncorhynchus tshawytscha*. Animal Genetics 33, 316-318.

[Olsen, B.J](http://search2.scholarsportal.info.cerberus.lib.uoguelph.ca/ids70/p_search_form.php?field=au&query=olsen+bj&log=literal&SID=d3be8fc344093fe8bc704e27125e3c97)., [Wilson, L.S](http://search2.scholarsportal.info.cerberus.lib.uoguelph.ca/ids70/p_search_form.php?field=au&query=wilson+ls&log=literal&SID=d3be8fc344093fe8bc704e27125e3c97)., [Kretschmer, J.E](http://search2.scholarsportal.info.cerberus.lib.uoguelph.ca/ids70/p_search_form.php?field=au&query=kretschmer+je&log=literal&SID=d3be8fc344093fe8bc704e27125e3c97)., [Jones, C.K](http://search2.scholarsportal.info.cerberus.lib.uoguelph.ca/ids70/p_search_form.php?field=au&query=jones+ck&log=literal&SID=d3be8fc344093fe8bc704e27125e3c97)., [Seeb, E.J](http://search2.scholarsportal.info.cerberus.lib.uoguelph.ca/ids70/p_search_form.php?field=au&query=seeb+ej&log=literal&SID=d3be8fc344093fe8bc704e27125e3c97)., 2000. [Characterization of 14 tetranucleotide microsatellite loci derived from sockeye salmon.](http://search2.scholarsportal.info.cerberus.lib.uoguelph.ca/ids70/view_record.php?id=3&recnum=0&SID=d3be8fc344093fe8bc704e27125e3c97) Molecular Ecology 9, 2185-2187.

Palti, Y., Danzmann, R., Rexroad, C.E. III., 2003. Characterization and mapping of 19 polymorphic microsatellite markers for rainbow trout (*Oncorhynchus mykiss*). Animal Genetics 34, 153-156.

Palti, Y., Fincham, M., Rexroad, C.E. III, 2002. Characterization of 38 polymorphic microsatellite markers for rainbow trout *(Oncorhynchus mykiss*). Molecular Ecology Notes 2, 449-452.

Rexroad, C.E. III, Lee, Y., Keele, J.W., Karamycheva, S., Brown, G., Koop, B., Gahr, S.A., Palti, Y., Quackenbush, J., 2003. Sequence analysis of a rainbow trout cDNA library and creation of a gene index. Cytogenetic Genome Research 102, 347-354.

Rexroad, C.E. III, Coleman, R.L., Hershberger, W.K., Killefer, J., 2002a. Eighteen polymorphic microsatellite markers for rainbow trout (*Oncorhynchus mykiss*). Animal Genetics 33, 76-78.

Rexroad, C.E. III, Coleman, R.L., Hershberger, W.K., Killefer, J., 2002b. Rapid communication: Thirty-eight polymorphic microsatellite markers for mapping in rainbow trout. Journal of Animal Science 80, 541-542.

Rexroad, C.E. III, Coleman, R.L., Gustafson, A.L., Hershberger, W.K., Killefer, J., 2002c. Development of Rainbow Trout Microsatellite Markers from Repeat Enriched Libraries. Marine Biotechnology 4, 12-16.

Rise, M.L., von Schalburg, K.R., Brown, G., Mawer, M.A., Devlin, R.H., Kuipers, N., Busby, M., Beetz-Sargent, M., Alberto, R., Gibbs, A.R., Hunt, P., Shukin, R., Zeznik, J.A., Nelson, C., Jones, S.R., Smailus, D.E., Jones, S.J., Schein, J.E., Marra, M.A., Butterfield, Y.S., Stott, J.M., Ng, S.H., Davidson, W.S., Koop, B., 2004. Development and application of a salmonid EST database and cDNA microarray: data mining and interspecific hybridization characteristics. Genome Research 14, 478-490.

Sakamoto, T., Danzmann, R., Gharbi, K., Howard, P., Ozaki, A., Khoo, S., Woram, R., Okamoto, N., Ferguson, M., Holm, L., Guyomard, R., Hoyheim, B., 2000. A microsatellite linkage map of rainbow trout (*Oncorhynchus mykiss*) characterized by large sex-specific difference in recombination rates. Genetics 155, 1331-1345.

Spies, I., Brasier, D.J., O'Reilly, P., Seamons, T.R., Bentzen, P., 2005. Development and characterization of novel tetra-, tri-, and dinucleotide microsatellite markers in rainbow trout (*Oncorhynchus mykiss*). Molecular Ecology Notes 5, 278-281.

Williamson, K.S., Cordes, J.F., May, B., 2002. Characterization of microsatellite loci in Chinook salmon (*Oncorhynchus tshawytscha*) and cross-species amplification in other salmonids. Molecular Ecology Notes 2, 17-19.

**Supplementary Table 2.** Linkage associations between genetic markers in the test parents (B5 and B11 = female parents while C1,

C2, and C4 = male parents). Linkage was assigned using a minimum LOD value of 3.0.

|  |  | **Parents** | | | | |
| --- | --- | --- | --- | --- | --- | --- |
| **Linkage Group** | **Markers** | **B5** | **B11** | **C1** | **C2** | **C4** |
| 1 | BX076085 | S | S | S | S | S |
|  |  |  |  |  |  |  |
| 2 | BX861650/i | - | - | U | U | U |
|  | OtsG43UCD/i | - | - | - | - | - |
|  | OMM1262/i | U | - | - | - | - |
|  | CA376300/i | U | S | U | U | U |
|  |  |  |  |  |  |  |
| 3 | OMM1230 | X | X | X | X | - |
|  | OMM1297 | X | - | - | - | X |
|  | BHMS206 | X | X | X | X | X |
|  | BX317661/i | X | X | X | X | X |
|  |  |  |  |  |  |  |
| 5 | BX318599/ii | S | - | - | S | S |
|  |  |  |  |  |  |  |
| 6 | OMM1302 | U | - | X | X | X |
|  | OMM1205 | - | - | X | X | X |
|  | OMM1359 | X | X | X | X | X |
|  | OMM1355 | X | X | X | X | X |
|  | OMM1082 | - | X | X | X | X |
|  |  |  |  |  |  |  |
| 7 | OMM1305 | X | X | X | S | X |
|  | OMM1034 | X | X | X | - | - |
|  | OMM1087 | U | U | X | - | X |
| Appendix 2 continued |  |  |  |  |  |  |
| **LG** | | **B5** | **B11** | **C1** | **C2** | **C4** |
| 8 | Omi134TUF | X | - | X | X | X |
|  | One112ADFG | X | - | X | X | X |
|  | Ots532NWFSC | - | - | X | X | X |
|  | OmyFGT12TUF | X | X | X | X | X |
|  | OMM1009 | X | - | X | X | X |
|  | BHMS415 | - | - | X | X | X |
|  | CA060381 | X | X | X | X | X |
|  | Clock | X | - | X | X | X |
|  | One114ADFG | X | X | U | - | U |
|  | OMM1304 | X | X | - | - | - |
|  |  |  |  |  |  |  |
| 9 | OmyRGT30TUF | U | - | - | X | X |
|  | CB497405 | X | - | S | X | X |
|  | CA376300/ii | X | S | - | X | X |
|  | OMM1262/ii | - | - | - | - | - |
|  |  |  |  |  |  |  |
| 10 | Omy1225UW | X | - | X | - | X |
|  | CR363293 | X | - | X | X | X |
|  | OMM1179 | - | S | X | X | - |
|  |  |  |  |  |  |  |
| 11 | Omy1011UW | X | - | - | - | X |
|  | OMM1315 | X | - | X | X | X |
|  | BX313739 | X | - | X | X | - |
|  | Ots515NWFSC | X | S | X | X | X |
|  |  |  |  |  |  |  |
| 12 | CA349039/ii | S | S | S | - | S |
|  |  |  |  |  |  |  |
|  |  |  |  |  |  |  |
|  |  |  |  |  |  |  |
| Appendix 2 continued |  |  |  |  |  |  |
| **LG** | | **B5** | **B11** | **C1** | **C2** | **C4** |
| 13 | OMM1216 | X | X | - | - | - |
|  | OmyRGT14TUF | X | - | X | X | X |
|  | OMM1321 | X | X | X | X | X |
|  |  |  |  |  |  |  |
| 14 | OMM1657/ii | S | - | U | S | X |
|  | OMM1134/i | - | S | - | - | - |
|  | BX080247 | - | - | U | - | X |
|  |  |  |  |  |  |  |
| 15 | OMM1260 | X | - | X | X | X |
|  | OMM1051 | X | X | X | X | X |
|  | OMM1036 | X | X | X | X | X |
|  | OMM1166 | X | - | X | - | - |
|  | OMM1175 | - | X | X | - | - |
|  | OmyRGT23TUF | X | - | - | - | - |
|  |  |  |  |  |  |  |
| 16 | CA345149 | X | U | - | X | X |
|  | BX867838 | - | U | S | X | X |
|  | CA349039/i | X | - | - | X | - |
|  |  |  |  |  |  |  |
| 17 | OMM1090 | S | U | X | U | U |
|  | BX305863 | - | U | X | U | U |
|  |  |  |  |  |  |  |
| 18 | Omi87TUF | - | - | X | X | - |
|  | OMM1311 | - | - | X | X | U |
|  | OmyRGT12TUF | - | U | X | X | - |
|  | OmyCosBTUF/i | - | U | - | - | - |
|  |  |  |  |  |  |  |
| 19 | Omy103INRA | U | U | X | X | X |
|  | OMM1025 | X | X | X | X | X |
| Appendix 2 continued |  |  |  |  |  |  |
| **LG** | | **B5** | **B11** | **C1** | **C2** | **C4** |
|  | CA058586 | - | X | - | X | X |
|  | BX298853 | X | X | - | X | - |
|  |  |  |  |  |  |  |
| 20 | BX882100/ii | - | X | - | - | - |
|  | OMM1257 | U | X | U | S | - |
|  | OMM1134/ii | - | - | - | - | - |
|  | OMM1657/i | U | - | U | - | S |
|  |  |  |  |  |  |  |
| 21 | BX302949 | S | S | U | U | U |
|  | OMM1059 | - | - | U | U | U |
|  |  |  |  |  |  |  |
| 22 | BX319197 | X | X | X | X | X |
|  | BX860095 | X | X | X | X | X |
|  |  |  |  |  |  |  |
| 23 | OMM1055 | - | U | - | - | U |
|  | OMM1097 | - | U | S | S | U |
|  |  |  |  |  |  |  |
| 24 | OMM1105 | X | X | X | X | X |
|  | BHMS377 | - | X | X | X | X |
|  | Omy27INRA | X | X | X | X | X |
|  | OmyRGT36TUF | X | X | X | - | - |
|  | Omy4DIAS | X | X | X | X | X |
|  | OMM1322 | X | X | X | X | X |
|  | OMM1320 | X | X | X | X | X |
|  |  |  |  |  |  |  |
| 25 | OMM1193 | S | U | U | - | U |
|  | Omy1552UW/i/ii | - | U | U | - | U |
|  |  |  |  |  |  |  |
| 26 | OMM1015 | U | X | S | X | - |
| Appendix 2 continued |  |  |  |  |  |  |
| **LG** |  | **B5** | **B11** | **C1** | **C2** | **C4** |
|  | CR375500 | U | X | - | X | S |
|  |  |  |  |  |  |  |
| 27 | BX863380 | - | X | X | - | - |
|  | CA374878 | S | X | X | X | X |
|  | BX073867 | - | U | X | X | X |
|  | OMM1228 | - | X | X | X | X |
|  |  |  |  |  |  |  |
| 29 | BX861650/ii | X | - | X | X | - |
|  | BX087664/ii | - | X | X | - | X |
|  | OtsG43UCD/ii | X | X | X | X | X |
|  | OMM1162 | X | X | - | - | X |
|  |  |  |  |  |  |  |
| 30 | BX079862 | X | S | X | X | X |
|  | OMM1186 | X | - | X | X | X |
|  |  |  |  |  |  |  |
| 31 | OmyRGT1TUF | U | U | U | U | X |
|  | BX318599/i | X | X | - | - | X |
|  | OMM1337 | X | X | U | U | U |
|  |  |  |  |  |  |  |

Linkage is denoted between polymorphic markers with an “X”. Monomorphic markers for which linkage data could not be obtained for

a particular parent is denoted by an “-”.

Markers denoted with a “U” were polymorphic, however remain unlinked to other markers genotyped in the linkage group indicated.

‘S’ indicates that only a single marker was genotyped in the linkage group indicated.
